# Fiscalin Derivatives as Potential Neuroprotective Agents

**DOI:** 10.3390/pharmaceutics14071456

**Published:** 2022-07-12

**Authors:** Sandra Barreiro, Bárbara Silva, Solida Long, Madalena Pinto, Fernando Remião, Emília Sousa, Renata Silva

**Affiliations:** 1 Associate Laboratory i4HB—Institute for Health and Bioeconomy, Faculty of Pharmacy, University of Porto, 4050-313 Porto, Portugal; barbarapolerisilva@gmail.com (B.S.); remiao@ff.up.pt (F.R.); 2 UCIBIO—Applied Molecular Biosciences Unit, Requimte, Laboratory of Toxicology, Department of Biological Sciences, Faculty of Pharmacy, University of Porto, 4050-313 Porto, Portugal; 3 Department of Bioengineering, Royal University of Phnom Penh, Russian Confederation Blvd., Phnom Penh 12156, Cambodia; solidachhann@gmail.com; 4 CIIMAR—Centro Interdisciplinar de Investigação Marinha e Ambiental, Terminal de Cruzeiros do Porto de Leixões, 4450-208 Matosinhos, Portugal; madalenakijjoa@gmail.com (M.P.); esousa@ff.up.pt (E.S.); 5 Laboratory of Organic and Pharmaceutical Chemistry, Department of Chemical Sciences, Faculty of Pharmacy, University of Porto, 4050-313 Porto, Portugal

**Keywords:** fiscalins, neurodegenerative diseases, cytotoxicity, P-glycoprotein, neuroprotection, neurodegeneration

## Abstract

Neurodegenerative diseases (ND) share common molecular/cellular mechanisms that contribute to their progression and pathogenesis. In this sense, we are here proposing new neuroprotection strategies by using marine-derived compounds as fiscalins. This work aims to evaluate the protective effects of fiscalin derivatives towards 1-methyl-4-phenylpyridinium (MPP^+^)- and iron (III)-induced cytotoxicity in differentiated SH-SY5Y cells, an in vitro disease model to study ND; and on P-glycoprotein (P-gp) transport activity, an efflux pump of drugs and neurotoxins. SH-SY5Y cells were simultaneously exposed to MPP^+^ or iron (III), and noncytotoxic concentrations of 18 fiscalin derivatives (0–25 μM), being the cytotoxic effect of both MPP^+^ and iron (III) evaluated 24 and 48 h after exposure. Fiscalins **1a** and **1b** showed a significant protective effect against MPP^+^-induced cytotoxicity and fiscalins **1b**, **2b**, **4** and **5** showed a protective effect against iron (III)-induced cytotoxicity. Fiscalins **4** and **5** caused a significant P-gp inhibition, while fiscalins **1c**, **2a**, **2b**, **6** and **11** caused a modest increase in P-gp transport activity, thus suggesting a promising source of new P-gp inhibitors and activators, respectively. The obtained results highlight fiscalins with promising neuroprotective effects and with relevance for the synthesis of new derivatives for the treatment/prevention of ND.

## 1. Introduction

Neurodegenerative diseases (ND) are debilitating conditions that affect the central nervous system (CNS), mostly related to the aging of the world population. ND are characterized by the dysfunction and loss of the neuronal structure and function, resulting in uncontrolled neuronal death that leads to a progressive decline in brain functions [1,2,3]. These diseases are correlated with a wide spectrum of clinical symptoms, involving cognitive decline, memory loss and/or the impairment of motor functions [1,2,4,5]. Furthermore, the severity of the ND symptoms gradually progresses alongside the disease development, leading to a reduced capability for independent living. With the increase in the average lifespan, the prevalence of CNS diseases tends to grow, alongside an increased impact of the serious effects of such diseases on the quality of life as well as an increased burden on healthcare systems worldwide [1,2,3].

ND diseases, including Alzheimer’s (AD) and Parkinson’s diseases (PD), have a complex etiology and pathogenesis, which explains why the existing drugs available for their treatment have limited efficacy [1,3]. The study of ND, and particularly PD, have been a priority, and countless reports suggest that the human neuroblastoma SH-SY5Y cell line holds several characteristics of dopaminergic neurons, with these cells being widely used for neuroprotection studies against neurotoxins capable of inducing characteristic features of PD [6,7,8,9,10]. Moreover, the SH-SY5Y cell line has been also used in studies of amyloid neurotoxicity, using both synthetic and endogenous Aβ peptides, with these cells also being useful for the study of AD [11]. In fact, the neuroblastoma SH-SY5Y cell line, a subline of the parental line SK-N-SH, obtained from a bone-marrow sympathetic adrenergic ganglial neuroblastoma, is able to produce and release catecholamines and can develop several neuronal properties, including neurite-like processes. Depending on the differentiation protocol, they can be differentiated into cholinergic, dopaminergic, and adrenergic cells [1,10,12,13]. For instance, retinoic acid stimulates the differentiation of SH-SY5Y cells into neuronal-like cells bearing extended neurites and expressing mature neuronal markers [10,12,14]. Thus, the SH-SY5Y cell line has been extensively used as a model for ND thanks to these particular features.

It is widely accepted that most ND share common molecular and cellular mechanisms that contribute to their progression and pathogenesis. These mechanisms include abnormal protein misfolding and aggregation, oxidative stress (OS), mitochondrial dysfunction, excitotoxicity, neuroinflammation and the dysregulation of calcium or some metals’ homeostasis [2,4,15]. Metal ions, particularly iron, have been related to ND pathogenesis and progression as key mediators of neurotoxicity, as their overaccumulation enhances OS through the Fenton Reaction; as well as leading to protein aggregation—including α-synuclein in PD; and beta-amyloid (Aβ) and tau hyperphosphorylation in AD, known hallmarks of these ND [16,17,18,19]. Moreover, and particularly for AD, the impairment of Aβ clearance pathways appears to also contribute to the progression of the disease [20,21,22]. P-Glycoprotein (P-gp), an efflux transporter from the adenosine triphosphate (ATP)-binding cassette (ABC) transporters family, mediates the efflux of several compounds, including Aβ peptides, and its reduced expression has been associated with Aβ accumulation and increased senile plaque formation, thus being related to the progression of the AD [20,22,23].

The currently available drug treatments for ND are predominantly directed to symptom relief, and often fail to reduce the progression of the disease [4,24,25,26]. Neuroprotection strategies and their related mechanisms are therefore of foremost importance to prevent or delay the neurodegeneration processes by interfering with pathophysiological mechanisms of neurodegeneration [5]. In this sense, we have been studying marine-derived compounds, as they present a broad spectrum of biological and pharmacological activities, contributing to the development of new chemical structures with promising therapeutic properties [15]. Fiscalins are a group of valine-derived alkaloids whose structures, closely related to fumiquinazolines, consist of an indolyl moiety connected to an anthranilic acid-derived tricyclic system [27]. These marine-derived compounds were firstly introduced in 1993 as substance P inhibitors, isolated from the fungus *Neosartorya fischeri* [28]. In fact, substance P inhibitors were already reported to have neuroprotective properties by using a 6-hydroxydopamine model of PD [29], and some fiscalin derivatives obtained by synthesis by Long et al. showed neuroprotective properties in SH-SY5Y cells treated with rotenone [30]. Moreover, other studies have shown that fiscalins also have antimicrobial [27,31,32,33], anticancer [27,34,35,36,37] and neuroprotective [30] properties. 

This work thus aims to evaluate the potential neuroprotective effect of a library of 18 fiscalin derivatives on differentiated SH-SY5Y cells, by using two different approaches: (i) protection against the cytotoxicity induced by two different stressors—1-methyl-4-phenylpyridinium (MPP^+^) (the active metabolite of the dopaminergic neurotoxin 1-methyl-4-phenyl-1,2,3,6-tetrahydropyridine (MPTP)) and Fe^3+^ (inducer of ferroptosis), in vitro disease models for the study of ND; and (ii) modulation (inhibition or activation) of P-glycoprotein transport activity (by Rhodamine 123 accumulation assay), an efflux pump of drugs (e.g., levodopa) and neurotoxins (as Aβ peptides), and remarkably important in ND treatment and disease progression.

Therefore, the obtained results highlight several fiscalins with promising neuroprotective effects as well as with relevance to the synthesis of new derivatives to be potentially used in the treatment and/or prevention of ND, since fiscalins **1a** and **1b** showed a significant protective effect against MPP^+^-induced cytotoxicity and fiscalins **1b**, **2b**, **4** and **5** showed a significant protective effect against iron (III)-induced cytotoxicity. Moreover, fiscalins **4** and **5** lead to P-gp inhibition, while fiscalins **1c**, **2a**, **2b**, **6** and **11** caused a modest but significant increase in P-gp transport activity, thus suggesting a promising source of new P-gp inhibitors and activators, respectively. 

## 2. Experimental Design, Materials and Methods

### 2.1. Materials

Reagents used in cell culture, including Dulbecco’s modified Eagle’s medium (DMEM) with high glucose, sodium bicarbonate, trypsin-ethylenediamine tetraacetic acid (EDTA) solution (0.25% trypsin/1 mM EDTA), retinoic acid (RA), 12-*O*-tetradecanoylphorbol-13-acetate (TPA), dimethyl sulfoxide (DMSO), Trizma^®^ base, rhodamine 123 (*RHO* 123), zosuquidar (ZOS), 1-methyl-4-phenylpyridinium iodide (MPP^+^ iodide), iron (III) chloride (FeCl_3_), nitrilotriacetic acid disodium salt (Na_2_NTA), nitrilotriacetic acid trisodium salt (Na_3_NTA), neutral red (NR) solution, sulforhodamine B (SRB) and resazurin (REZ) were obtained from Sigma-Aldrich (Taufkirchen, Germany). Antibiotic mixture (10,000 U/mL penicillin, 10,000 μg/mL streptomycin) and nonessential amino acids (NEAA) were obtained from Biochrom (Berlin, Germany). Triton™ X-100 detergent solution was acquired from Thermo Fisher Scientific (Waltham, MA, USA). Heat-inactivated fetal bovine serum (FBS), Hanks’ balanced salt solution (HBSS) with or without calcium and magnesium [HBSS (+/+) or HBSS (−/−), respectively], and phosphate buffer solution with or without calcium and magnesium [PBS (+/+) or PBS (−/−), respectively] were obtained from Gibco (Paisley, UK). All the reagents used were of analytical grade or of the highest grade available.

### 2.2. Synthesis of Fiscalins

**Figure 1:**
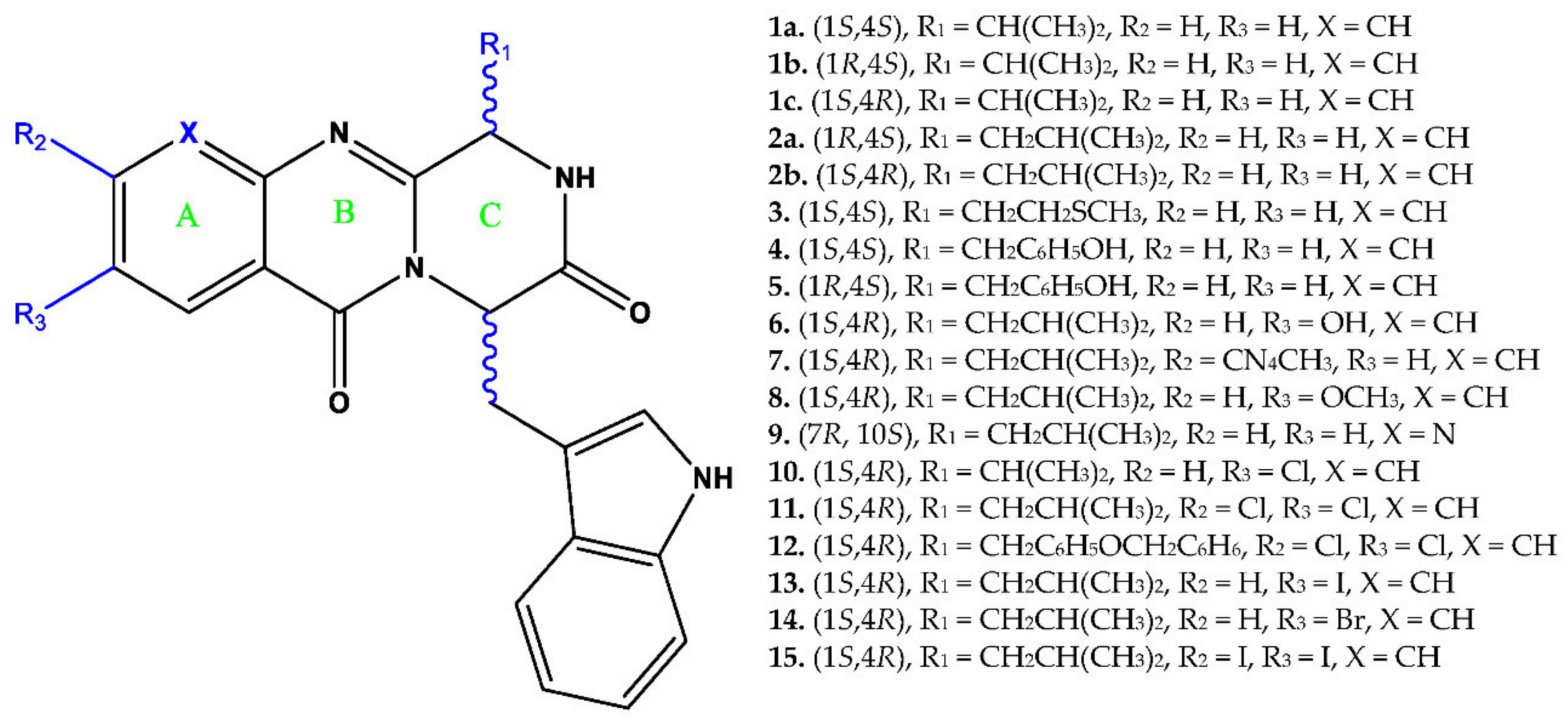
Chemical structure of the fiscalin derivatives under research in the present study.

Fiscalins are indole-containing pyrazino[2,1-b] quinazoline-3,6-diones [38], and to be evaluated in this study, eighteen fiscalin derivatives were synthetized (Figure 1).

**Scheme 1:**
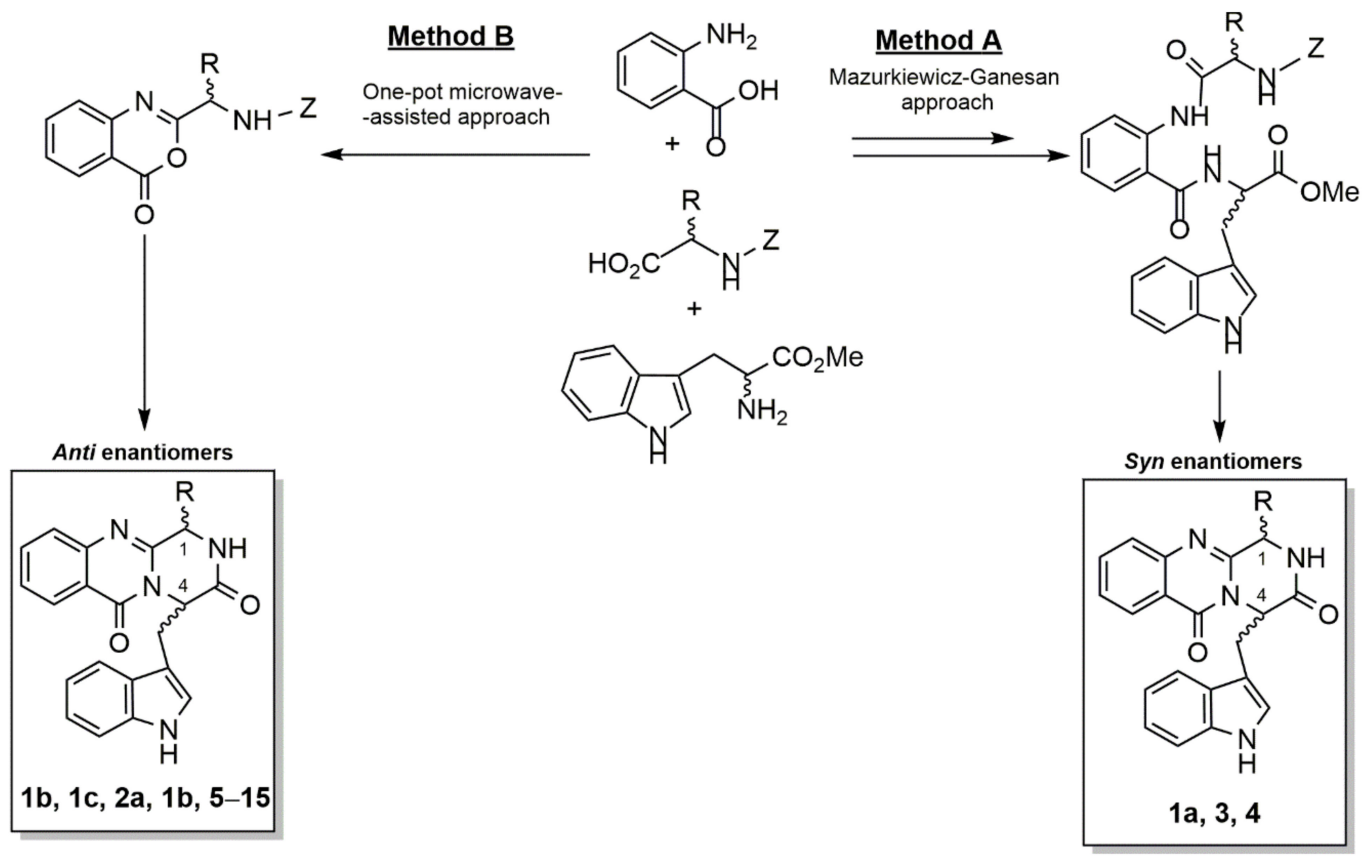
Synthetic pathway to obtain fiscalins derivatives **1a**, **1b**, **1c**, **2a**, **2b** and **3**–**15**.

Two distinct approaches were developed and optimized over the last few years toward the synthesis of the fiscalins’ scaffold [30,38]. Compound **1a** was previously synthesized in our laboratory, via Mazurkiewicz–Ganesan approach, consisting of coupling of linear tripeptides follow by isomerization of 4-imino-4*H*-3,1-benzoxozines to obtain the corresponding quinazolin-4-ones of **1a**, **3** and **4** (Scheme 1 Method A) by removing a protecting group. Derivatives **1b**, **1c**, **2a**, **2b** and **5**–**15** were also previously obtained using a microwave-assisted multicomponent one-step polycondensation of amino acids [30,32,33,38,39,40]. The procedure was summarized as a condensation of *N*-protected α-amino acids with anthranilic acids under mild heating with triphenylphosphite (PhO)_3_P, generating the intermediate benzoxazine-4-one, followed by flash microwave irradiation with tryptophan ester to yield the desired products (Scheme 1 Method B).

Indole-coquinazolinone-3,6-(4H)-diones compounds **1a**, **1b**, **1c**, **2a**, **2b**, **3**–**15** were previously synthesized and used without further purification after purity assessment [30,32,33,38,39,40]: (1*S*,4*S*)-4-(1H-Indol-3-ylmethyl)-1-isopropyl-2H-pyrazino[2,1-b] quinazolin-3,6-(1H, 4H)-dione (**1a**), (1*R*,4*S*)-4-(1H-Indol-3-ylmethyl)-1-isopropyl-2H-pyrazino[2,1-b]quinazoline-3,6-(1H,4H)-dione (**1b**), (1*S*,4*R*)-4-(1H-Indol-3-ylmethyl)-1-isopropyl-2H-pyrazino[2,1-b]quinazoline-3,6-(1H,4H)-dione (**1c**), (1*R*,4*S*)-4-(1H-Indol-3-ylmethyl)-1-isobutyl-2H-pyrazino[2,1-b]quinazoline-3,6-(1H,4H)-dione (**2a**), (1*S*,4*R*)-4-(1H-Indol-3-ylmethyl)-1-isobutyl-2H-pyrazino[2,1-b]quinazoline-3,6-(1H,4H)-dione (**2b**) [38]. (1*S*,4*R*)-4-((1H-indol-3-yl)methyl)-1-(2-(methylthio)ethyl)-1,2-dihydro-6H-pyrazino[2,1-b]quinazoline-3,6(4H)-diones (**3**), (1*S*,4*S*)-4-((1H-indol-3-yl)methyl)-1-(4-hydroxybenzyl)-1,2-dihydro-6H-pyrazino[2,1-b]quinazoline-3,6(4H)-dione (**4**), (1*R*,4*S*)-4-((1H-indol-3-yl)methyl)-1-(4-hydroxybenzyl)-1,2-dihydro-6H-pyrazino[2,1-b]quinazoline-3,6(4H)-dione (**5**) [30], (1*S*,4*R*)-4-((1H-indol-3-yl)methyl)-8-hydroxy-1-isobutyl-1,2-dihydro-6H-pyrazino[2,1-b]quinazoline-3,6(4H)-dione (**6**), (1*S*,4*R*)-4-((1H-indol-2-yl)methyl)-1-isobutyl-9-(1-methyl-1H-tetrazol-5-yl)-1,2-dihydro-6H-pyrazino [2,1-b]quinazoline-3,6(4H)-dione (**7**), (1*S*,4*R*)-4-((1H-indol-3-yl)methyl)-1-isobutyl-8-methoxy-1,2-dihydro-6H-pyrazino[2,1-b]quinazoline-3,6(4H)-dione (**8**), (7*R*,10*S*)-7-((1H-indol-3-yl)methyl)-10-isobutyl-9,10-dihydro-5H-pyrazino[1,2-a]pyrido[2,3-d]pyrimidine-5,8(7H)-dione (**9**) [33], (1*S*,4*R*)-4-((1H-indol-3-yl)methyl)-8-chloro-1-isopropyl-1,2-dihydro-6H-pyrazino[2,1-b]quinazoline-3,6(4H)-dione (**10**), (1*S*,4*R*)-4-((1H-indol-3-yl)methyl)-8-chloro-1-isobutyl-1,2-dihydro-6H-pyrazino[2,1-b]quinazoline-3,6(4H)-dione (**11**) [32], (1*S*,4*R*)-4-((1H-indol-3-yl)methyl)-1-(4-(benzyloxy)benzyl)-8,10-dichloro-1,2-dihydro-6H-pyrazino[2,1-b]quinazoline-3,6(4H)-dione (**12**) [30], (1*S*,4*R*)-4-((1H-indol-3-yl)methyl)-8-iodo-1-isopropyl-1,2-dihydro-6H-pyrazino[2,1-b]quinazoline-3,6(4H)-dione (**13**), (1*S*,4*R*)-4-((1H-indol-3-yl)methyl)-8-bromo-1-isopropyl-1,2-dihydro-6H-pyrazino[2,1-b]quinazoline-3,6(4H)-dione (**14**), (1*S*,4*R*)-4-((1H-indol-3-yl)methyl)-8,10-diiodo-1-isobutyl-1,2-dihydro-6H-pyrazino[2,1-b]quinazoline-3,6(4H)-dione (**15**) [32].

A 50 mM stock solution of each fiscalin derivative (Figure 1) was prepared in DMSO and all stock solutions were stored at −20 °C and freshly diluted on the day of the experiment, in fresh cell culture medium (ensuring that DMSO concentration did not exceed 0.1% of the exposure media).

### 2.3. SH-SY5Y Cell Culture and Differentiation

SH-SY5Y cells (ATCC, United States of America) were routinely cultured in 25 cm^2^ flasks using DMEM with 4.5 g/L glucose, supplemented with 10% heat-inactivated FBS, 1% antibiotic mixture (100 U/mL of penicillin and 100 μg/mL of streptomycin) and 1% NEAA. Cells were maintained in a 5% CO_2_—95% air atmosphere, at 37 °C, and the medium was changed every 2–3 days. When 80–90% confluence was reached, the cell cultures were passaged by trypsinization (0.25% trypsin/1 mM EDTA). In all experiments, the cells were seeded in 96- or 24-well plates at a density of 25,000 cells/cm^2^ and used 6 days after seeding. In order to obtain cells with a dopaminergic neuronal phenotype, SH-SY5Y cells were differentiated as previously described [41,42]. Briefly, SH-SY5Y cells were seeded in complete DMEM medium containing 10 μM RA, and cultured for 3 days at 37 °C. After 3 days, 80 nM of TPA was added to the cultures, and cells were cultured for another 3 days at 37 °C. The cells used in all experiments were taken between the 21st and 27th passages.

### 2.4. Fiscalin Cytotoxicity

Fiscalin cytotoxicity was evaluated in differentiated SH-SY5Y cells, by the neutral red (NR) uptake, resazurin (REZ) reduction and sulforhodamine B (SRB) binding assays, 24 and 48 h after exposure. For that purpose, SH-SY5Y cells were seeded in 96-well plates at a density of 25,000 cells/cm^2^ and differentiated through the RA and TPA differentiation protocol. Six days after seeding, differentiated SH-SY5Y cells were exposed to fiscalins (0–50 μM), prepared in fresh cell culture medium. Triton™ X-100 (0.1%) was used as positive control for all the cytotoxicity experiments. At least four independent experiments were performed, in triplicate.

#### 2.4.1. Neutral Red Uptake Assay

The NR uptake assay is based on the ability of viable cells to incorporate and bind the NR dye in the lysosomes, providing a quantitative analysis of the number of viable cells in culture [43,44]. At 24 or 48 h after exposure to the tested fiscalins (**1a**, **1b**, **1c**, **2a**, **2b** and **3**–**15**), the cell culture medium was removed and the cells were washed with HBSS (+/+), followed by the addition of fresh cell culture medium containing 50 μg/mL NR, and incubation, at 37 °C, in a humidified 5% CO_2_—95% air atmosphere, for 60 min. After 60 min incubation with NR, the cell culture medium was removed, and the dye that was only absorbed by viable cells extracted with a lysis buffer [absolute ethyl alcohol/distilled water (1:1) with 5% acetic acid]. The absorbance was then measured at 540 nm, in a multiwell plate reader (PowerWaveX BioTek Instruments, Winooski, VT, USA). The results were expressed as percentage of NR uptake relatively to that of the control cells (0 µM). At least four independent experiments were performed, in triplicate.

#### 2.4.2. Resazurin Reduction Assay

The REZ reduction assay, also known as alamarBlue assay, is based on the conversion of the blue nonfluorescent dye resazurin to the pink fluorescent resorufin by mitochondrial reductases and other cytoplasmic enzymes, such as diaphorases. Consequently, the quantity of the produced resorufin is related to the number of metabolically viable cells in culture [43,45]. After exposure to the tested fiscalins (**1a**, **1b**, **1c**, **2a**, **2b** and **3**–**15**) for 24 or 48 h, the cell culture medium was removed and the cells were washed with HBSS (+/+), followed by the addition of fresh cell culture medium containing 10 μg/mL REZ. The cells were then incubated, at 37 °C, in a humidified 5% CO_2_—95% air atmosphere, for 60 min. The fluorescence was read afterwards in a multiwell plate reader (PowerWaveX BioTek Instruments, Winooski, VT, USA), using an excitation and emission wavelengths of 560 nm and 590 nm, respectively. The results were expressed as percentage of REZ reduction relative to that of the control cells (0 µM). At least four independent experiments were performed, in triplicate.

#### 2.4.3. Sulforhodamine B Binding Assay

The sulforhodamine B (SRB) protein-staining assay is an in vitro cytotoxicity assay developed to measure all the cellular protein content. This assay is based on the dye’s ability to bind to basic amino acids of cellular proteins under mild acidic conditions. Consequently, the total protein mass, which is related to the number of cells in culture, can be determined by spectrophotometry [43,46]. After exposure to the tested fiscalins (**1a**, **1b**, **1c**, **2a**, **2b** and **3**–**15**) for 24 or 48 h, the cell culture medium was removed, the cells were washed with HBSS (+/+) and fixed overnight, at −20 °C, with a methanolic solution of 1% acetic acid (*v*/*v*). The fixing medium was then removed, replaced by 0.05% SRB solution (prepared in 1% acetic acid), followed by incubation, at 37 °C, for 60 min. After incubation, the SRB solution was removed, and the cells were washed with 1% acetic acid (*v*/*v*) to remove the unbound dye. The plates were left to air-dry at room temperature and the bound SRB was subsequently extracted with a Tris base solution (10 mM, pH 10.5). The absorbance was measured, at 540 nm, in a multiwell plate reader (PowerWaveX BioTek Instruments, Winooski, VT, USA). The results were expressed as percentage of SRB binding relative to that of the control cells (0 µM). At least four independent experiments were performed, in triplicate.

### 2.5. P-Glycoprotein Modulation Studies

Changes in the expression and/or activity of ABC transporters at the blood–brain barrier (BBB) have been associated with many neurological diseases, as well as with the pharmacoresistance to central-nervous-system (CNS) acting drugs [22]. The modulation of the activity of ABC transporters, including P-gp, can be identified through the intracellular accumulation of a probe substrate in the presence of a test compound [47]. Consequently, an increased intracellular accumulation of a probe substrate in the presence of a given compound indicates that the test compound is an inhibitor of the efflux transporter [48,49]. On the opposite, a decreased intracellular accumulation of a probe substrate in the presence of a test compound suggests its potential for the efflux transporter activation [50,51,52]. A fluorescent P-gp substrate widely used in accumulation assays is rhodamine 123 (*RHO* 123), which allows for the direct evaluation of P-gp activity by measuring *RHO* 123 intracellular fluorescence [47,51,52,53,54,55,56]. Accordingly, in the present work, the fiscalins’ effect on P-gp transport activity was evaluated in a multiwell plate reader, by the *RHO* 123 accumulation assay, using *RHO* 123 (10 μM) as a P-gp fluorescent substrate, and ZOS (5 μM), as a specific third-generation P-gp inhibitor. Although the *RHO* 123 and ZOS concentrations were selected based in previous studies performed by our group, those studies were performed in different cell lines (Caco-2, SW480 and RBE4 cells [51,52,53,54,56,57]). Therefore, preceding the evaluation of the effects of fiscalin derivatives on P-gp activity, the cytotoxicity of both *RHO* 123 (0–10 μM) and ZOS (0–5 μM) was evaluated in differentiated SH-SY5Y cells, by the REZ reduction, NR uptake, and SRB binding assays, upon 24 h of exposure. Only the 24 h timepoint was selected, since in most of the accumulation assays performed to assess the potential modulatory effect of new compounds on P-gp activity, shorter periods of incubation with both the P-gp substrate and inhibitor are required [51,52,53,54,56,57,58]. These cytotoxicity results are present in the Supplementary Materials, available on the journal page (Figure S1). In addition, and although the experimental conditions used to evaluated the fiscalins’ effects on P-gp activity were based on previous studies [51,53,54,55,56,57], the *RHO* 123 concentration, as well as the duration of the incubation with the P-gp fluorescent substrate, were optimized using differentiated SH-SY5Y cells. Furthermore, these optimization results are available in the Supplementary Materials (Figure S2).

#### *RHO* 123 Accumulation Assay in the Presence of Fiscalins

For the in vitro evaluation of the effects of fiscalins on P-gp transport activity, SH-SY5Y cells were seeded in 24-well plates at a density of 25,000 cells/cm^2^, following the differentiation protocol with RA and TPA, as previously mentioned. Six days after seeding, the cell culture medium was removed, and the cells were exposed to noncytotoxic concentrations of fiscalins **1a**, **1b**, **1c**, **2a**, **2b** and **3**–**11** (0–25 μM), in the presence and in the absence of the P-gp inhibitor (ZOS). Briefly, after differentiation, SH-SY5Y cells were submitted to the following procedures:

1. *RHO* 123 accumulation under normal conditions (NA): the cells were exposed to the tested fiscalins (0–25 μM), prepared in HBSS (+/+), for 30 min, and further incubated with *RHO* 123 (10 μM), also prepared in HBSS (+/+), for 90 min, at 37 °C, in a humidified 5% CO_2_—95% air atmosphere. Control cells were only exposed to the P-gp fluorescent substrate.
2. *RHO* 123 accumulation in the presence of the P-gp inhibitor (inhibited accumulation, IA): differentiated SH-SY5Y cells were simultaneously exposed to the tested fiscalins and to the specific P-gp inhibitor, ZOS (5 μM), both prepared in HBSS (+/+). After 30 min, *RHO* 123 (10 μM), prepared in HBSS (+/+), was added, and the cells were further incubated for 90 min, at 37 °C, in a humidified 5% CO_2_—95% air atmosphere. Control cells were only exposed to the P-gp fluorescent substrate and to the P-gp inhibitor.

Each of the previously mentioned exposure conditions (NA and IA, in the presence or in the absence of the tested compounds) were performed in triplicate. After the incubation period, the cells were washed twice with HBSS (+/+) and lysed with 0.1% Triton™ X-100 for 30 min at room temperature and in the absence of light. After lysis, the intracellular *RHO* 123 fluorescence was measured in a multiwell plate reader (PowerWave-X, BioTek Instruments, Winooski, VT, USA) at excitation/emission wavelengths of 485/528 nm, respectively, and expressed as fluorescence intensity (*FI*). Four independent experiments were performed, in triplicate.

P-gp activity was then evaluated through the ratio between the *FI* after accumulation of *RHO* 123 in the presence of the P-gp inhibitor (*IA*) and the *FI* of the accumulation of *RHO* 123 in the absence of the P-gp inhibitor (*NA*) (Equation (1)):

(1) $$RHO\;123\;Accumulation=\frac{FI\;of\;RHO\;123\;accumulation\;under\;P-gp\;inhibition\;\left(IA\right)}{FI\;of\;RHO\;123\;accumulation\;under\;normal\;conditions\;\left(NA\right)}$$

The results were expressed as percentage of control cells (0 μM fiscalins).

When the P-gp activity increases, the amount of *RHO* 123 that is pumped out of the cell is higher, and consequently the fluorescence intensity decreases as a consequence of the decrease in the *RHO* 123 intracellular content. Therefore, as the fluorescent substrate is pumped out of the cells during the accumulation period due to the pump activation, the *FI_NA_* will decrease, and the *FI_IA_*/*FI_NA_* ratio will be higher. Conversely, when P-gp is inhibited, its activity is reduced, leading to an increased intracellular *RHO* 123 accumulation, and therefore, to a higher fluorescence intensity. Consequently, the ratio *FI_IA_*/*FI_NA_* will be lower, since a higher fluorescence intensity under normal conditions (*FI_NA_*) will be detected, because of the decreased *RHO* 123 efflux.

### 2.6. Evaluation of Fiscalins’ Neuroprotective Effects

To evaluate the potential neuroprotective effects of the fiscalin derivatives under study, differentiated SH-SY5Y cells were exposed to two different stressors: MPP^+^-iodide, the active metabolite of the dopaminergic neurotoxin MPTP and iron (III), through ferric nitrilotriacetate reagent (FeNTA).

#### 2.6.1. Fiscalins’ Protective Effects against MPP^+^-Induced Cytotoxicity

SH-SY5Y cells were seeded at a density of 25,000 cells/cm^2^ into 96-well plates, following the RA and TPA differentiation protocol previously mentioned. Six days after seeding, the cells were exposed to fiscalins (0–25 μM) prepared in fresh cell culture medium. After 30 min of preincubation, MPP^+^ (0–1500 μM) was added and the cells were then incubated at 37 °C in a humidified 5% CO_2_—95% air atmosphere, for 24 or 48 h. Following the incubation period, MPP^+^ cytotoxicity was evaluated by the NR uptake assay, as described above, since it was previously shown to be the most sensitive method to detect MPP^+^-induced cytotoxicity (data not shown). At least four independent experiments were performed, in triplicate. For each experiment, a fresh stock solution of MPP^+^ (10 mM) was prepared (protected from light) in PBS (+/+), diluted afterwards in fresh cell culture medium and used immediately.

#### 2.6.2. Fiscalins’ Protective Effects against Iron (III)-Induced Cytotoxicity

To evaluate the potential neuroprotective effects of the fiscalin derivatives under study against iron (III)-induced cytotoxicity, differentiated SH-SY5Y were exposed to ferric nitrilotriacetate (FeNTA), a ferric (Fe^3+^) iron aggressor. Initially, the NTA (250 mM, pH 7.4) solution was prepared by combining a nitrilotriacetic acid disodium salt (Na_2_NTA) solution (100 mM) and a nitrilotriacetic acid trisodium salt (Na_3_NTA) solution (100 mM) until pH = 7 was achieved. Iron (III) chloride was then added to the NTA to obtain the FeNTA solution. A fresh stock solution of FeNTA (100 mM) was prepared for each experiment (protected from light) and was firstly incubated for 15 min before use to ensure the complete formation of the ferric oxidation form before addition to the cell cultures, and then diluted in fresh cell culture medium [17,59]. SH-SY5Y cells were seeded at a density of 25,000 cells/cm^2^ into 96-well plates, following the RA and TPA differentiation protocol previously mentioned. Six days after seeding, the cells were exposed to fiscalins (0–25 μM) prepared in fresh cell culture medium. After 30 min of pre-incubation, FeNTA (0–1500 μM) was added, and the cells were then incubated, at 37 °C, in a humidified 5% CO_2_—95% air atmosphere, for 24 or 48 h. Following the incubation period, FeNTA cytotoxicity was evaluated by the NR uptake assay, as described in the previous sections. At least four independent experiments were performed, in triplicate.

### 2.7. Statistical Analysis

GraphPad Prism 8 for Windows (GraphPad Software, San Diego, CA, USA) was used to perform all statistical calculations. Three tests were performed to evaluate the normality of the data distribution: the KS normality test, D’Agostino and Pearson omnibus normality test and Shapiro–Wilk normality test. For data with parametric distribution, a one-way ANOVA was used to perform the statistical comparisons, followed by Dunnett’s multiple comparisons test. Statistical comparisons between groups in experiments with two variables were made using two-way ANOVA, followed by Holm–Šídák’s multiple comparisons test. Details of the performed statistical analysis are described in the figure legend. In all cases, differences were considered to be significant for *p* values lower than 0.05.

## 3. Results

### 3.1. Fiscalin Cytotoxicity

The cytotoxicity of 18 fiscalins, **1a**, **1b**, **1c**, **2a**, **2b**, **3** to **15** (0–50 μM), were evaluated in differentiated SH-SY5Y cells, by the NR uptake, REZ reduction and SRB binding assays, 24 and 48 h after exposure, in order to select the nonharmful fiscalins and their respective noncytotoxic concentrations to be further used in the subsequent neuroprotection and P-gp modulation studies.

**Figure 2:**
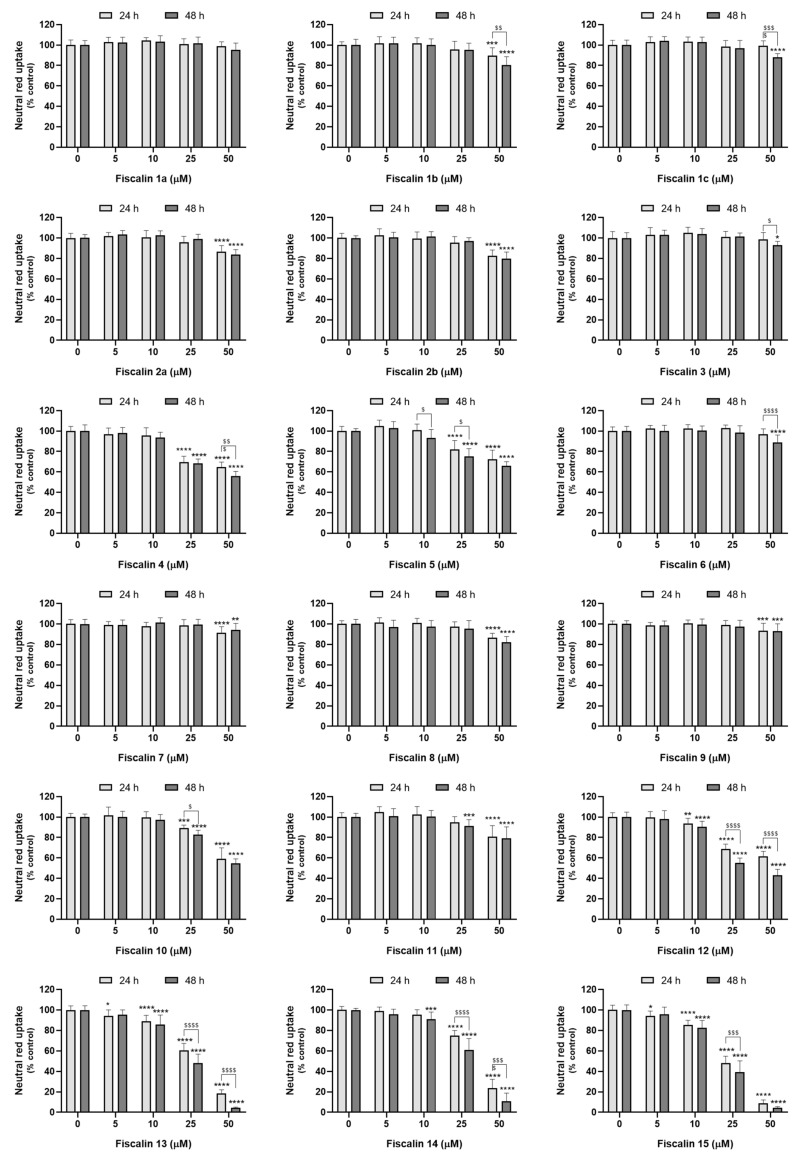
Fiscalins (0–50 µM) cytotoxicity towards differentiated SH-SY5Y cells, evaluated by the neutral red uptake assay, 24 and 48 h after exposure. Results are presented as mean ± SD from at least four independent experiments, performed in triplicate. Statistical comparisons were made using two-way ANOVA, followed by Tukey’s multiple comparisons test (at each timepoint, for comparisons between concentrations) or by Sidak’s multiple comparisons test (for each concentration, for comparisons between timepoints). In all cases, *p* values < 0.05 were considered significant [* *p* < 0.05; ** *p* < 0.01; *** *p* < 0.001; **** *p* < 0.0001 versus control (0 µM), at each timepoint; ^$^ *p* < 0.05; ^$$^ *p* < 0.01; ^$$$^ *p* < 0.001; ^$$$$^ *p* < 0.0001 24 h versus 48 h, at each concentration]. Fiscalin concentrations vs. timepoints interaction for fiscalin **1a**: F (4,117) = 0.6818, *p* = 0.6059; **1b**: F (4,123) = 2.572, *p* = 0.0411; **1c**: F (4,228) = 9.889, *p* < 0.0001; **2a**: F (4,122) = 1.239, *p* = 0.2979; **2b**: F (4,124) = 1.027, *p* = 0.3964; **3**: F (4,120) = 1.374, *p* = 0.2471; **4**: F (4,118) = 3.226, *p* = 0.0149; **5**: F (4,124) = 1.735, *p* = 0.1465; **6**: F (4,168) = 3.463, *p* = 0.0095; **7**: F (4,170) = 0.9703, *p* = 0.4253; **8**: F (4,170) = 1.234, *p* = 0.2985; **9**: F (4,168) = 0.1091, *p* = 0.9792; **10**: F (4,119) = 1.082, *p* = 0.3684; **11**: F (4,208) = 0.7257, *p* = 0.5753; **12**: F (4,169) = 20.60, *p* < 0.0001; **13**: F (4,169) = 13.96, *p* < 0.0001; **14**: F (4,170) = 8.422, *p* < 0.0001; **15**: F (4,170) = 3.963, *p* = 0.0042.

In the NR uptake assay (Figure 2), no significant effects were detected after exposure to fiscalin **1a** at any of the tested concentrations, for both timepoints. Additionally, no significant effects were also detected 24 h after exposure to fiscalins **1c**, **3** and **6** at any of the tested concentrations. However, an increased and significant decrease in the NR uptake was detected 48 h after exposure to the highest concentration of these fiscalins (**1c** and **6**: *p* < 0.0001; **3:** *p* < 0.05)**.** For fiscalins **1b**, **2a**, **2b**, **7**, **8** and **9** a significant decrease in the NR uptake, although small, was detected 24 h after exposure to 50 μM, an effect also verified at the 48 h timepoint. For fiscalin **11**, a significant decrease in the NR uptake (*p* < 0.0001) was observed 24 h after exposure to the highest tested concentration (50 μM), an effect intensified at the 48 h timepoint, with significant effects also being observed for 25 μM of fiscalin **11** (*p* < 0.001). Additionally, for fiscalins **4**, **5** and **10,** a significant decrease in the NR uptake was detected 24 h after exposure to the 25 (**4** and **5**: *p* < 0.0001; **10:** *p* < 0.001) and 50 μM (**4**, **5** and **10**: *p* < 0.0001) concentrations, and the effect was slightly intensified after 48 h of exposure to these fiscalins. On the opposite, the most cytotoxic compounds detected were fiscalins **12, 13, 14** and **15**, with significant decreases in the NR uptake detected for concentrations equal or higher that 10 μM, at both timepoints. For fiscalin **12**, NR uptake significantly decreased to 93.7% (*p* < 0.01), 68.8% (*p* < 0.0001) and 61.8% (*p* < 0.0001), 24 h after exposure to 10, 25 and 50 μM of fiscalin **12**, and to 90.3% (*p* < 0.0001), 55.0% (*p* < 0.0001) and 43.1% (*p* < 0.0001), 48 h after exposure to 10, 25 and 50 μM of fiscalin **12**, respectively. For fiscalin **14**, NR uptake significantly decreased to 74.9% (*p* < 0.0001) and 23.8% (*p* < 0.0001), 24 h after exposure to 25 and 50 μM of fiscalin **14**, and to 91.1% (*p* < 0.001), 60.9% (*p* < 0.0001) and 10.9% (*p* < 0.0001), 48 h after exposure to 10, 25 and 50 μM of fiscalin **14**, respectively. Additionally, for fiscalins **13** and **15**, 24 and 48 h after exposure, a significant decrease in the NR uptake (*p* < 0.0001) was verified for concentrations equal or higher than 10 μM, at both timepoints.

Similar results were detected in the REZ reduction (Supplementary Materials, Figure S3) and in the SRB binding (Supplementary Materials, Figure S4) assays, with a concentration- and time-dependent cytotoxic effect being observed for several of the tested compounds, comparatively to the control cells (0 µM).

By the overall analysis of the cytotoxicity assays’ results, the SRB binding assay proved to be the less sensitive method, while the NR uptake assay was shown to be the most sensitive, with this one being further used in the neuroprotection studies. Importantly, compounds **12**, **13**, **14** and **15** were the most cytotoxic among the tested fiscalins, with a substantial and significant decrease in cell viability, as detected by all three cytotoxicity assays. Considering their high cytotoxicity, these four compounds were excluded from the subsequent studies.

The remaining compounds showed no significant cytotoxicity for concentrations below or equal to 25 µM, being the concentrations 10 and 25 µM the selected ones to be further used in the neuroprotection and P-gp modulation studies. However, compounds **4**, **5**, **10** and **11** demonstrated to be cytotoxic for concentrations equal or above 25 µM, particularly for the 48 h exposure timepoint. In this sense, for fiscalins **4** and **5**, upon 24 h of exposure, and for compounds **4**, **5**, **10** and **11** upon 48 h of exposure, the concentration selected was only 10 µM. Noteworthy, the noncytotoxic concentrations selected (10 and 25 µM) have already been reported for in vitro studies with similar fiscalin derivatives [30,32,34,38,60].

### 3.2. P-Glycoprotein Modulation Studies

P-gp activity was evaluated in differentiated SH-SY5Y cells after incubation of the tested fiscalin derivatives, **1a**, **1b**, **1c**, **2a**, **2b**, **3**–**11** (0–25 μM) in the presence and absence of ZOS (5 μM), by performing a *RHO* 123 accumulation for a period of 90 min, aiming to evaluate the potential immediate effects of the fiscalin derivatives on P-gp transport activity as a result of a direct inhibition or activation of this pump.

**Figure 3:**
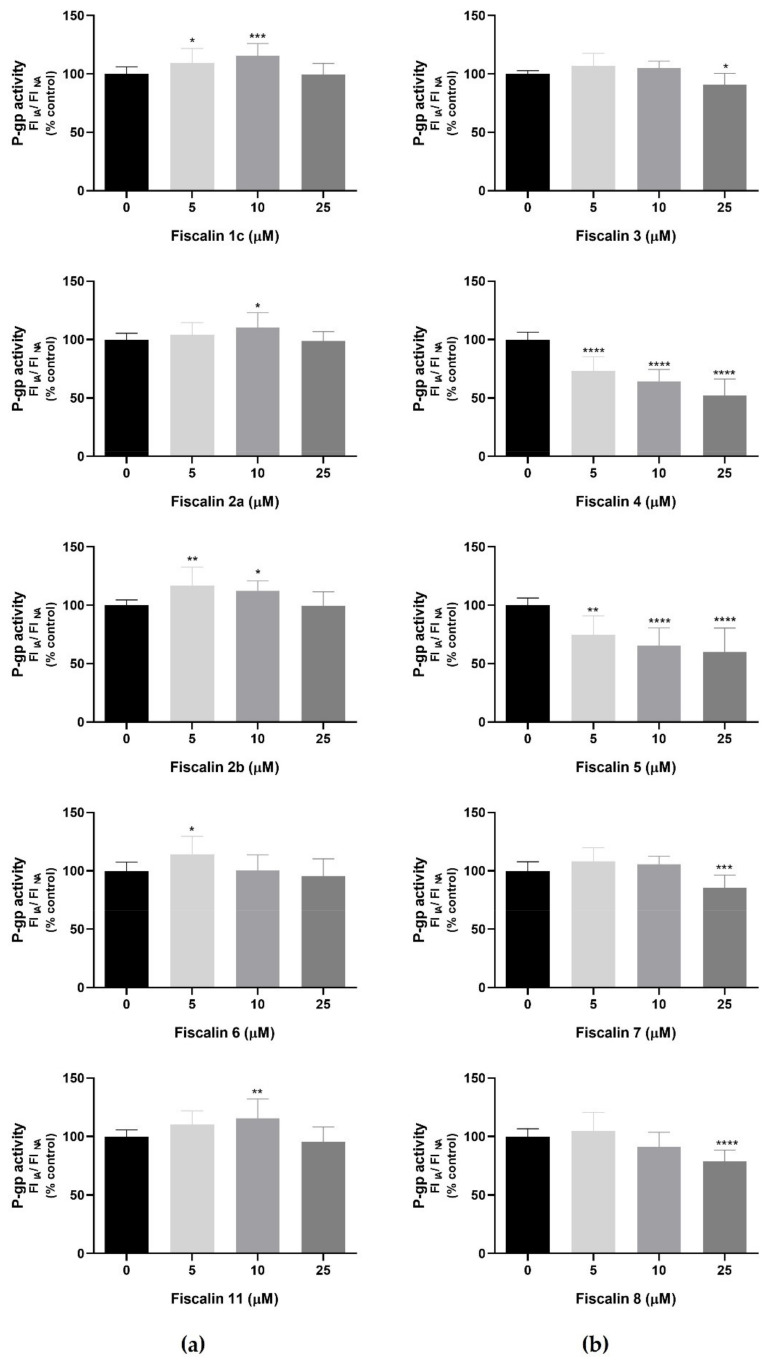
Evaluation of the fiscalins (0–25 μM) potential for P-glycoprotein activation (**a**) and inhibition (**b**). P-glycoprotein activity was evaluated by fluorescence spectroscopy in differentiated SH-SY5Y cells exposed to the tested fiscalins (0–25 μM) during the 90 min incubation period with the fluorescent substrate (10 μM *RHO* 123). Results are presented as mean ± SD from four independent experiments, performed in triplicate. Statistical comparisons were made using the parametric method of one-way ANOVA, followed by Dunnett’s multiple comparisons test [* *p* < 0.05; ** *p* < 0.01; *** *p* < 0.001; **** *p* < 0.0001 vs. control (0 μM)].

According to the obtained results, no significant effect on P-gp activity was observed for fiscalins **1a**, **1b**, **9** and **10**, at any of the tested concentration and when compared to control cells (0 μM) (Supplementary Materials, Figure S5). Fiscalins **2a** and **11** were shown to slightly but significantly increase P-gp activity at the 10 μM concentration (110.2%, *p* < 0.05, and 115.5%, *p* < 0.01, after exposure to 10 μM of compounds **2a** and **11**, respectively), while fiscalin **6** caused a slight but significant (*p* < 0.05) increase in P-gp activity at 5 μM (114.3%, when compared to control cells). In addition, fiscalins **1c** and **2b** slightly but significantly increased P-gp activity at 5 and 10 μM (P-gp transport activity significantly increased to 109.4%, *p* < 0.05, and 115.3%, *p* < 0.001, and to 117.0%, *p* < 0.01, and 112.3%, *p* < 0.05, after exposure to 5 and 10 μM of fiscalins **1c** and **2b**, respectively). These results, as shown in Figure 3a, although not significant for all the tested concentrations (0–25 μM), indicate a P-gp activation effect.

Fiscalins **3**, **7** and **8** produced a small but significant decrease in P-gp activity (Figure 3b), when compared to control cells, although only at the 25 μM concentration (P-gp activity significantly decreased to 90.9%, *p* < 0.05, 85.3%, *p* < 0.001, and 78.9%, *p* < 0.0001, after exposure to 25 μM of compounds **3**, **7** and **8**, respectively). Noteworthy, and as observed in Figure 3b, fiscalins **4** and **5**, at all of the tested concentrations (0–25 μM), caused a significant and concentration-dependent decrease in the P-gp activity, demonstrating the ability to reduce *RHO* 123 efflux, by consequently increasing the intracellular accumulation of the P-gp fluorescent substrate. In fact, P-gp transport activity significantly decreased to 73.2% (*p* < 0.0001), 64.3% (*p* < 0.0001), and 52.0% (*p* < 0.0001) of control cells after exposure to 5, 10 and 25 μM of fiscalin **4**; and to 74.6% (*p* < 0.01), 65.6% (*p* < 0.0001), and 60.0% (*p* < 0.0001) after exposure to 5, 10 and 25 μM of fiscalin **5**.

### 3.3. Fiscalins Neuroprotective Effects

#### 3.3.1. MPP^+^ as an Agent for Chemical-Induced Cytotoxicity—Evaluation of Fiscalins’ Protective Effects

To evaluate the potential protective effect of fiscalins towards MPP^+^-induced cytotoxicity, differentiated SH-SY5Y cells were simultaneously exposed to MPP^+^ (0–1500 μM) and to fiscalins **1a**, **1b**, **1c**, **2a**, **2b**, **3**–**11**. The cytotoxic effect of MPP^+^, in the presence and absence of the fiscalins, was further evaluated 24 and 48 h after exposure by the NR uptake assay. According to the cytotoxicity assays, fiscalins **1a**, **1b**, **1c**, **2a**, **2b**, **3**, **6**, **7**, **8** and **9** were tested at 10 and 25 μM for 24 and 48 h of exposure. For both timepoints, fiscalins **4** and **5** were only tested at 10 μM, given the observed cytotoxicity towards differentiated SH-SY5Y cells. For the same reason, fiscalins **10** and **11**, due to their cytotoxicity profile, were tested at 10 and 25 μM in the studies involving 24 h of exposure to MPP^+^, and only at 10 μM for studies with 48 h of exposure.

According to the obtained data, three different patterns of effects were observed after 24 h of fiscalin incubation: in some concentrations, fiscalins **1a** and **1b** significantly protected differentiated SH-SY5Y cells against MPP^+^-induced cytotoxicity; fiscalins **1c**, **2b**, **4**, **5** and **7** did not affect MPP^+^-induced cytotoxicity at any concentration (Supplementary Materials, Figure S6); and finally, fiscalins **2a**, **3**, **6**, **8**, **9**, **10** and **11** were found to increase MPP^+^-induced cytotoxicity towards differentiated SH-SY5Y cells (Supplementary Materials, Figure S6).

In the same way, 48 h after incubation, fiscalin **1a** preserved its significant protective effect against MPP^+^-induced cytotoxicity; fiscalins **2a** and **6** slightly but significantly protected differentiated SH-SY5Y cells against MPP^+^-induced cytotoxicity; fiscalins **1c**, **1b**, **3** and **7** did not affect MPP^+^-induced cytotoxicity at any concentration (Supplementary Materials, Figure S7); and fiscalins **2b**, **4**, **5**, **8**, **9**, **10** and **11** were found to increase MPP^+^-induced cytotoxicity towards differentiated SH-SY5Y cells (Supplementary Materials, Figure S7).

**Figure 4:**
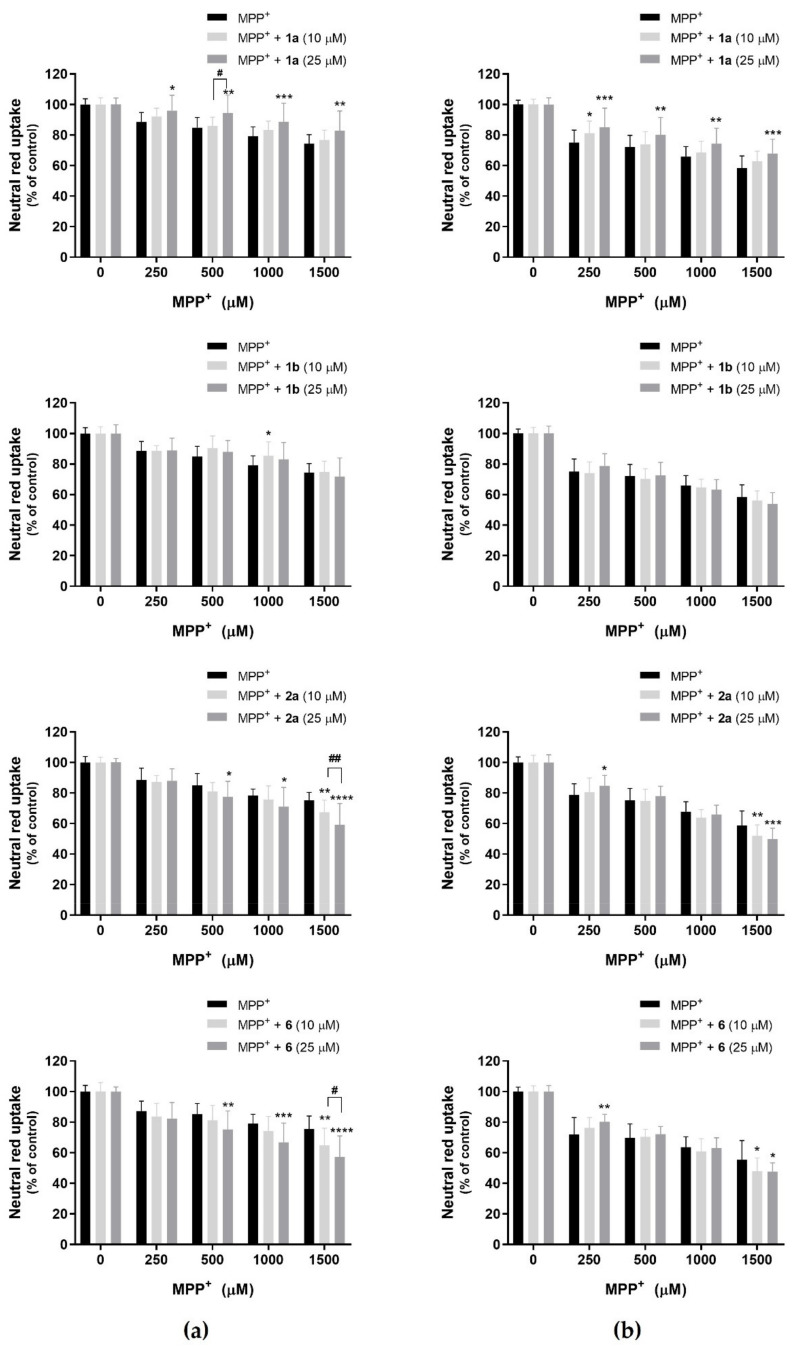
MPP^+^ (0–1500 µM) cytotoxicity evaluated in differentiated SH-SY5Y cells, in the presence or absence of the most promising fiscalins (**1a**, **1b**, **2a** and **6**) (10 and 25 µM), evaluated by the neutral red uptake assay, 24 (**a**) and 48 h (**b**) after exposure. Results are presented as mean ± SD from 4 independent experiments, performed in triplicate. Statistical comparisons were made using two-way ANOVA, followed by Holm–Šídák’s multiple comparisons test. In all cases, *p* values < 0.05 were considered significant [* *p* < 0.05; ** *p* < 0.001; *** *p* < 0.001; **** *p* < 0.0001 versus MPP^+^ alone; ^#^ *p* < 0.05; ^##^ *p* < 0.001 at each MPP^+^ concentration for comparisons between the 10 and 25 µM concentrations of the tested compounds]. Treatment with MPP^+^ vs. fiscalin concentration interaction (24 h after exposure) for fiscalins **1a**: F (8,213) = 1.312, *p* = 0.2387; **1b**: F (8,216) = 1.045, *p* = 0.4033; **2a**: F (8,194) = 2.882, *p* = 0.0047; **6**: F (8,214) = 2.692, *p* = 0.0077. Treatment with MPP^+^ vs. fiscalin concentration interaction (48 h after exposure) for fiscalins **1a**: F (8,276) = 1.398, *p* = 0.1972; **1b**: F (8,277) = 1.140, *p* = 0.3364; **2a**: F (8,318) = 3.848, *p* = 0.0002; **6**: F (8,205) = 2.587, *p* = 0.0103.

In fact, fiscalin **1a**, at 25 μM, showed a significant protective effect against MPP^+^-induced cytotoxicity upon 24 h of exposure (NR uptake significantly increased to 95.9%, *p* < 0.05, 94.5%, *p* < 0.01, 88.8%, *p* < 0.001, and 82.9%, *p* < 0.01, 24 h after exposure to 250, 500, 1000 and 1500 μM of MPP^+^ in the presence of 25 μM of fiscalin **1a**, and when compared to 88.6%, 84.8%, 79.1% and 74.4% for MPP^+^ alone), as shown in Figure 4a. Upon 48 h of exposure to MPP^+^, fiscalin **1a** again showed a significant protective effect against MPP^+^-induced cytotoxicity, as observed in Figure 4b, since in the presence of 25 μM of fiscalin **1a**, a significant increase in the NR uptake was observed for all the tested MPP^+^ concentrations (NR uptake significantly increased to 85.0%, *p* < 0.001, 80.0%, *p* < 0.01, 74.3%, *p* < 0.01, and 67.9%, *p* < 0.001, 48 h after exposure to 250, 500, 1000 and 1500 μM of MPP^+^ in the presence of 25 μM of fiscalin **1a**, when compared to 75.0%, 72.1%, 65.8% and 58.4% for MPP^+^ alone). Additionally, when MPP^+^ was incubated in the presence of 10 μM of fiscalin **1a,** a significant increase in the NR uptake was also observed, but only for 250 μM of MPP^+^ (81.3%, *p* < 0.05, upon exposure to 250 μM of MPP^+^ for 48 h in the presence of 10 μM of fiscalin **1a**, when compared to 75.0% observed for MPP^+^ alone).

For fiscalin **1b**, at 10 μM, a minimal but significant (*p* < 0.05) increase in the NR uptake was observed for the 1000 μM concentration of MPP^+^, when compared to MPP^+^ alone, indicating the capacity for this fiscalin to also reduce MPP^+^-induced cytotoxicity. However, fiscalin **1b** did not retain the significant protective effect 48 h after exposure to MPP^+^.

For fiscalin **2a**, at 25 μM, a significant decrease in the NR uptake was observed for concentrations equal or above 500 µM of MPP^+^ (NR uptake significantly decreased to 77.6%, *p* < 0.05, 71.1%, *p* < 0.05, and 59.3%, *p* < 0.0001, 24 h after exposure to 500, 1000 and 1500 μM MPP^+^ in the presence of 25 μM compound **2a**, when compared to 85.1%, 78.4% and 75.3% for 500, 100 and 1500 μM MPP^+^ alone). A significant reduction (*p* < 0.01) in the NR uptake was also detected 24 h after exposure for 1500 µM of MPP^+^ in the presence of compound **2a** at 10 μM and when compared to MPP^+^ alone, as shown in Figure 4a. In a similar way, 24 h after exposure, the MPP^+^ cytotoxic effect was increased in the presence of fiscalin **6** (NR uptake significantly decreased to 75.2%, *p* < 0.01, 66.9%, *p* < 0.001, and 57.1%, *p* < 0.0001, 24 h after exposure to 500, 1000 and 1500 μM of MPP^+^ in the presence of 25 μM of compound **6**, when compared to 85.3%, 79.0% and 75.5% for 500, 100 and 1500 μM of MPP^+^ alone). Furthermore, for the highest concentration tested of MPP^+^, a significant reduction (*p* < 0.01) in the NR uptake was also detected in the presence of compound **6** at 10 μM (65.0%), and when compared to MPP^+^ alone (75.5%) (Figure 4a). Nevertheless, fiscalins **2a** and **6** showed a conflicting behavior towards MPP^+^-induced cytotoxicity in differentiated SH-SY5Y cells, after 48 h of exposure (Figure 4b). In fact, a significant reduction in the NR uptake was observed for the 1500 µM of MPP^+^ in the presence of fiscalins **2a** and **6** at 10 and 25 μM. However, in the presence of fiscalins **2a** and **6** at 25 μM, a slight but significant increase in the NR uptake was observed for the 250 μM MPP^+^ concentration (NR uptake significantly increased to 84.7%, *p* < 0.05, 48 h after exposure to 250 µM of MPP^+^ in the presence of 25 μM fiscalin **2a**, when compared to 78.7% for MPP^+^ alone; and to 80.2%, *p* < 0.01, 48 h after exposure to 250 µM MPP^+^ in the presence of 25 μM of fiscalin **6**, when compared to 72.1% for MPP^+^ alone).

On the other hand, for fiscalins **1c** and **7**, at 10 and 25 μM, no significant differences on the NR uptake were detected for any of the tested concentrations of MPP^+^ at either timepoint (Supplementary Materials, Figures S6 and S7). These results suggest that these compounds neither have a protective effect towards MPP^+^-induced cytotoxicity nor further increase its cytotoxic effect in differentiated SH-SY5Y cells. For fiscalins **2b**, **4** and **5**, at both tested concentrations, no significant differences on the NR uptake were detected for any of the MPP^+^ concentrations at 24 h of exposure (Supplementary Materials, Figure S6), but after 48 h, these fiscalins were found to significantly increase MPP^+^-induced cytotoxicity towards differentiated SH-SY5Y cells for concentrations equal or above 1000 μM of MPP^+^ (Supplementary Materials, Figure S7). Moreover, fiscalins **3**, **8**, **9**, **10** and **11** were found to increase MPP^+^-induced cytotoxicity towards differentiated SH-SY5Y cells, 24 (Supplementary Materials, Figure S6) and 48 h (Supplementary Materials, Figure S7) after exposure, particularly for fiscalins **10** and **11**, whose MPP^+^-induced cytotoxicity was significantly higher in their presence, at noncytotoxic concentrations, with the cytotoxic effect being significantly higher for 25 μM of these two compounds, and when compared to 10 μM, showing a concentration-dependent worsening of the MPP^+^ cytotoxic effect.

#### 3.3.2. FeNTA as an Agent for Iron-Induced Cytotoxicity—Evaluation of Fiscalins’ Protective Effects

Iron cytotoxic effect was evaluated in differentiated SH-SY5Y cells by the NR uptake assay, upon 24 and 48 h of exposure to FeNTA (0–1500 µM), in the presence of fiscalins **1a**, **1b**, **1c**, **2a**, **2b**, **3**–**11**. Again, and accordingly to the previous cytotoxicity assays performed in this work, fiscalins **1a**, **1b**, **1c**, **2a**, **2b**, **3**, **6**, **7**, **8**, and **9** were tested at 10 and 25 μM, for 24 and 48 h of exposure, given the lack of significant cytotoxicity towards differentiated SH-SY5Y cells. However, for both timepoints, fiscalins **4** and **5** were only tested at 10 μM, given the observed cytotoxicity towards differentiated SH-SY5Y cells observed at higher concentrations. For the same reason, fiscalins **10** and **11** were tested at 10 and 25 μM in the studies involving 24 h of exposure to FeNTA, and only at 10 μM for studies with 48 h of exposure.

**Figure 5:**
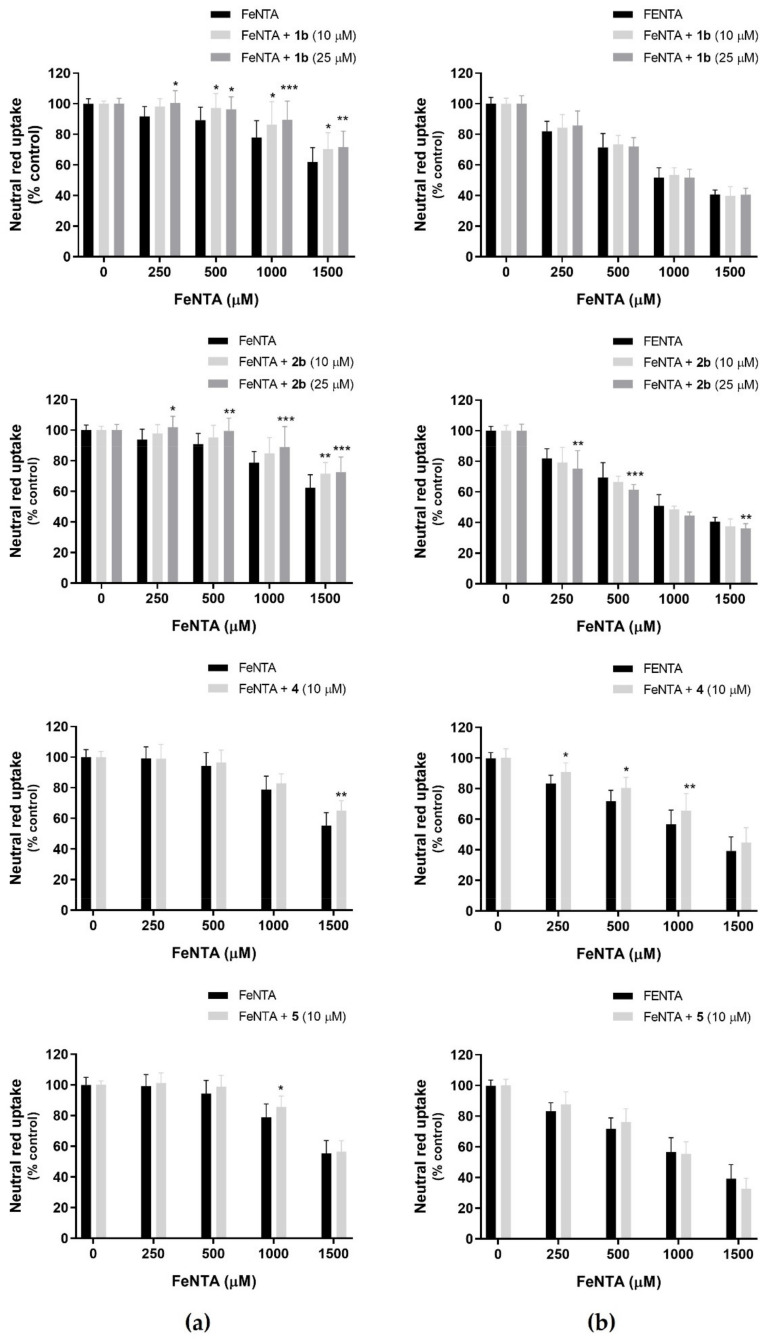
FeNTA (0–1500 µM) cytotoxicity evaluated in differentiated SH-SY5Y cells, in the presence or absence of the most promising fiscalins (**1b**, **2b**, **4** and **5**) (10 and 25 µM), evaluated by the neutral red uptake assay, 24 (**a**) and 48 h (**b**) after exposure. Results are presented as mean ± SD from 4 independent experiments, performed in triplicate. Statistical comparisons were made using two-way ANOVA, followed by Holm–Šídák’s multiple comparisons test. In all cases, *p* values < 0.05 were considered significant [* *p* < 0.05; ** *p* < 0.001; *** *p* < 0.001 versus FeNTA alone]. Treatment with FeNTA vs. fiscalin concentration interaction (24 h after exposure) for fiscalins **1b**: F (8,229) = 1.324, *p* = 0.2322; **2b**: F (8,212) = 1.490, *p* = 0.1621; **4**: F (4,165) = 2.258, *p* = 0.0651; **5**: F (4,165) = 1.087, *p* = 0.3647. Treatment with FeNTA vs. fiscalin concentration interaction (48 h after exposure) for fiscalins **1b**: F (8,243) = 0.4560, *p* = 0.8860; **2b**: F (8,250) = 1.098, *p* = 0.3648; **4**: F (4,149) = 1.568, *p* = 0.1859; **5**: F (4,154) = 2.854, *p* = 0.0256.

As observed along with MPP^+^-induced cytotoxicity, three different patterns of fiscalins’ effects were observed after 24 h of exposure to FeNTA: fiscalins **1b, 2b, 4** and **5** had a protective effect for some of the concentrations tested (Figure 5a); fiscalins **1a**, **1c**, **2a, 3, 6**, **7, 9, 10** and **11** did not affect the NR uptake at any tested concentration (data showed in Supplementary Materials, Figure S8); and compound **8** was found to significantly increase FeNTA-induced cytotoxicity, at both tested concentrations (Figure S8).

In the same way, 48 h after incubation, fiscalin **4** preserved its significant protective effect against FeNTA-induced cytotoxicity (Figure 5b); fiscalins **1a**, **3**, **6**, **10** and **11**, as observed for the 24 h incubation period, did not affect FeNTA-induced cytotoxicity at any concentration (Supplementary Materials, Figure S9); and, fiscalins **1c**, **2a**, **2b**, **7**, **8** and **9** were found to increase FeNTA-induced cytotoxicity towards differentiated SH-SY5Y cells (Supplementary Materials, Figure S9).

In fact, in the presence of fiscalin **1b,** at 10 μM, a significant increase in the NR uptake was observed for concentrations equal or above 500 μM of FeNTA. NR uptake significantly increased to 97.2% (*p* < 0.05), 86.5% (*p* < 0.05) and 70.2% (*p* < 0.05), 24 h after exposure to 500, 1000 and 1500 μM of FeNTA in the presence of fiscalin **1b**, when compared to 89.3%, 78.0% and 62.0% observed for FeNTA alone. Additionally, a significant increase in the NR uptake was also observed for all of the tested concentrations of FeNTA (250–1500 μM) in the presence of fiscalin **1b** at 25 μM, as shown in Figure 5a (100.4%, *p* < 0.05, 96.3%, *p* < 0.05, 89.5%, *p* < 0.001, and 71.7%, *p* < 0.01, 24 h after exposure to 250, 500, 1000 and 1500 μM of FeNTA in the presence of 25 μM of fiscalin **1b**, when compared to 91.7%, 89.3%, 78.0% and 62.0% observed for FeNTA alone). Unfortunately, the significant protective effect in the presence of fiscalin **1b** was not observed 48 h after exposure to FeNTA, as shown in Figure 5b.

In the presence of fiscalin **2b**, at 10 μM, a significant increase (*p* < 0.01) in the NR uptake was observed only for 1500 μM FeNTA, and when compared to FeNTA alone. Additionally, a significant increase in the NR uptake was observed for all tested FeNTA concentrations in the presence of fiscalin **2b** at 25 μM, and when compared to FeNTA alone, 24 h after exposure, as shown in Figure 5a (101.9%, *p* < 0.05, 99.5%, *p* < 0.01, 89.0%, *p* < 0.001, and 72.6%, *p* < 0.001, for 250, 500, 1000 and 1500 μM of FeNTA in the presence of 25 μM of fiscalin **2b**, when compared to 93.8%, 91.0%, 78.8% and 62.4% observed for 250, 500, 1000 and 1500 μM of FeNTA alone). However, 48 h after exposure to FeNTA, in the presence of fiscalin **2b**, the protective effect completely disappeared, and the FeNTA cytotoxic effect was slightly increased in the presence of 25 μM of fiscalin **2b**, as shown in Figure 5b.

In the presence of fiscalin **4**, at 10 μM, a significant increase (*p* < 0.01) in the NR uptake was observed for the 1500 μM of FeNTA (65.0%), as shown in Figure 5a, and when compared to FeNTA alone (55.4%). Moreover, upon 48 h of exposure to FeNTA in the presence of fiscalin **4** at 10 μM, a significant increase in the NR uptake was also observed for 250, 500 and 1000 μM of FeNTA, when compared to FeNTA alone. In fact, NR uptake slightly but significantly increased to 90.9% (*p* < 0.05), 80.5% (*p* < 0.05) and 65.6% (*p* < 0.01), 48 h after exposure to 250, 500 and 1000 μM of FeNTA in the presence of 10 μM of fiscalin **4**, and when compared to 83.3%, 71.8% and 56.6% observed for 250, 500 and 1000 μM of FeNTA alone (Figure 5b).

Additionally, 24 h after exposure (Figure 5a), a significant increase in the NR uptake (*p* < 0.05) was observed for 1000 μM of FeNTA in the presence of fiscalin **5** at 10 μM, and when compared to FeNTA alone. However, 48 h after exposure, no protective or increased cytotoxic effect was observed for FeNTA in the presence of fiscalin **5** (Figure 5b).

Adding to these results, for fiscalins **1a**, **3**, **6**, **10** and **11** (10 and 25 μM), no significant differences were detected on NR uptake for any of the tested FeNTA concentrations (0–1500 μM) either 24 or 48 h after exposure (Supplementary Materials, Figures S8 and S9, respectively). Nevertheless, iron (III)-induced cytotoxicity significantly increased in the presence of some of the tested fiscalins, namely fiscalins **1c**, **2a**, **7**, **8** and **9**, particularly for the 48 h timepoint (Supplementary Materials, Figure S9).

## 4. Discussion

Marine-derived compounds, including fiscalins, present important biological activities that might provide valuable tools in drug discovery and development, and are also considered promising alternatives, particularly for the treatment of neurodegenerative diseases [15,30]. Therefore, with this work we aimed at discovering new fiscalin derivatives with promising neuroprotective properties. To select noncytotoxic working concentrations, differentiated SH-SY5Y cells were initially exposed to the 18 fiscalin derivatives and their cytotoxicity were assessed by the NR uptake, REZ reduction and SRB binding assays, 24 and 48 h after exposure. According to the obtained results, and given the lack of significant effects, concentrations equal to or below 25 μM were selected as noncytotoxic working concentrations to be used in the neuroprotection and P-gp modulation studies. Of all the cell viability methods used, the SRB binding assay proved to be the less sensitive method, while the NR uptake assay showed to be the most sensitive, with this one being further used in neuroprotection studies. Given their cytotoxicity profile, fiscalins **12**, **13**, **14** and **15** were excluded from further experiments. Considering structure–cytotoxicity relationships, it was possible to assess that the presence of halogens in the fiscalin scaffold (Figure 1) clearly generates derivatives with higher cytotoxicity (which is the case of these compounds).

One of the known hallmarks of AD is the accumulation of Aβ peptides and subsequent formation of senile plaques, leading to neuronal degeneration. An imbalance between the production of Aβ peptides and their elimination leads to Aβ peptide accumulation. Within the BBB, P-gp, as one of the transporters responsible for the Aβ peptides’ elimination, has an important role in Aβ detoxification, and consequently in the pathogenesis of AD [61,62]. In vivo studies have suggested an increased Aβ accumulation, in the presence of P-gp inhibitors, a consequence of the decrease in P-gp activity [63]. Additionally, Aβ accumulation was shown to be reduced in the presence of P-gp inducers [64]. In the present work, some fiscalin derivatives, namely fiscalins **1c**, **2a**, **2b**, **6**, and **11**, showed a modest but significant increase in P-gp activity. Despite the short incubation period, the activation of this pump mediated by these compounds demonstrates their potential for modulating P-gp transport activity, and thus for increasing Aβ efflux, suggesting a promising source of new P-gp activators, with potential to increase Aβ detoxification, in the scope of AD treatment and/or prevention.

Despite the role of P-gp in Aβ detoxification, this transporter is also known to prevent the accumulation of therapeutic drugs within the brain, by actively pumping these drugs back into the blood stream, limiting their therapeutic efficacy [54,65]. For instance, studies have shown that P-gp inhibitors may increase the therapeutic efficacy of anticancer drugs. In fact, in vitro anticancer activity of doxorubicin was improved when in the presence of inhibitors of this efflux pump, some of which were marine-derived compounds, including fiscalin derivatives [37]. Moreover, some commonly used PD drugs, such as levodopa or dopamine agonists, are known to be P-gp substrates [66,67]. Therefore, P-gp inhibitors may ultimately have potential to improve pharmacological treatments for CNS diseases. Overall, regarding the present work, the results obtained for fiscalins **3**, **7**, **8**, and, particularly, **4** and **5** clearly demonstrate the inhibitory ability of these compounds towards the P-gp transport activity, therefore suggesting a promising source of new inhibitors, with potential to be used in CNS diseases, including ND, as adjuvants for drugs that are substrates of this efflux pump, possibly increasing their therapeutic efficacy.

Given the structure–activity relationship of these compounds, although compounds **1a**, **1b** and **1c** present the same structure, the different stereochemistry revealed to be important for the modulation of P-gp activity. For instance, compound **1c** with *R* stereochemistry at C-4 (ring C, Figure 1), was able to activate the P-gp, even though compounds **1a** and **1b**, both with *S* stereochemistry at C-4, showed no activity on this efflux pump. Additionally, the insertion of a chloro substituent in the anthranilic portion in fiscalin **1c** (fiscalin **10**) led to an absence of P-gp modulatory activity. Differently, compounds **2a** and **2b**, also stereoisomers, were both shown to activate P-gp, probably due to the presence of an isobutyl group in position 1, instead of an isopropyl group, which is the case of fiscalins **1a**, **1b** and **1c**. Moreover, and taking into account fiscalin **2b**, by adding one hydroxyl group (fiscalin **6**) or two chloro substituents (fiscalin **11**) in the aromatic ring of the anthranilic portion of its structure (ring A, Figure 1), no modification on P-gp activation was observed. Furthermore, fiscalins **3**–**5**, which have larger substituents at C-1, showed to inhibit P-gp. Particularly for fiscalins **4** and **5**, both with a phenolic ring in this position, which presented a significant concentration-dependent P-gp inhibition. In fact, given the structure–activity relationships, the presence of a phenolic ring attached to the fiscalin scaffold, adding to their long carbonated chain, could be related to the P-gp inhibitory effect of fiscalins **4** and **5**, both with *R* stereochemistry at C-4. Additionally, compounds **7** and **8**, compounds with larger substituents in the aromatic ring of the anthranilic portion of the fiscalin scaffold (ring A, Figure 1), showed to also inhibit P-gp, although compounds with smaller substituents in this position (-OH, compound **6**) were able to activate this efflux pump.

The loss of dopaminergic neurons within the *substantia nigra* is one of the pathological hallmarks of PD. This disease pathogenesis has been experimentally replicated, in several in vivo and in vitro models, by using the neurotoxin 1-methyl-4-phenylpyridinium (MPP^+^), the active metabolite of 1-methyl-4-phenyl-2,3,6-tetrahydropyridine (MPTP). This toxic compound is capable of severely damaging neuronal cells, inducing cell death, leading to a syndrome that closely resembles PD [6,7,8,68]. MPP^+^ is responsible for the inhibition of complex I of the mitochondrial electron transport chain, leading to reactive oxygen species (ROS) production and ATP depletion, altering the mitochondrial membrane potential, resulting in apoptosis and dopaminergic neuron death [7,8,68,69,70]. Furthermore, MPP^+^-induced dopaminergic cytotoxicity has been commonly used to investigate potential neuroprotective agents, in vitro and in vivo [69].

Several natural compounds, including marine-derived compounds, have shown promising neuroprotective properties towards MPP^+^-induced cytotoxicity with potential effectiveness for ND treatments, particularly for PD [7,60,71,72]. For instance, marine fungal metabolites such as neoechinulin A and xyloketal B have shown neuroprotective effects against MPP^+^-induced cytotoxicity in PC12 cells [73,74]. Additionally, neoechinulin A has also demonstrated to ameliorate the cytotoxic effect caused by rotenone, another chemical agent able to reproduce PD features [75]. According to the reported data, the indole alkaloid from marine fungi, neoechinulin A, significantly protected PC12 cells against the cytotoxicity induced by the Parkinson’s disease-inducing neurotoxin, MPP^+^, by ameliorating the downstream events that result from mitochondrial complex I inactivation. However, the blocking of rotenone-induced cell death was only observed upon cotreatment with neoechinulin A, and not upon neoechinulin A pretreatment before rotenone. Nevertheless, the mechanism(s) underlying the cytoprotective effects remains unclear [75]. Moreover, a study has already demonstrated fiscalins’ potential protective effects upon rotenone-induced cytotoxicity [30]. In fact, fiscalin derivatives, including the synthetic derivatives of the secondary metabolites fumiquinazoline G and fiscalin B (compound **1b**), and *epi*-**3** showed more than 25% protection against rotenone-induced cytotoxicity towards SH-SY5Y cells, as evaluated by the MTT assay, suggesting their neuroprotection capacity [30]. Adding the results obtained in the present work, fiscalin derivatives, although with limited studies available, seem to be promising neuroprotective agents. Indeed, the discovery of fiscalins A-C [28] as substance P inhibitors was also previously described as a promising novel neuroprotective therapy on early stages of PD, in an in vivo intrastriatal 6-hydroxydopamine model [29], anticipating this scaffold with potential neuroprotective properties that could be relevant for the treatment of ND, namely PD.

In our study, upon 24 h of exposure, two of the tested fiscalin derivatives (**1a** and **1b**) caused a significant increase in the NR uptake, suggesting a potential protective effect against MPP^+^-induced cytotoxicity. However, after 48 h of exposure, only compound **1a** maintained a significant protective effect. Moreover, 48 h after exposure, fiscalins **2a** and **6** did also show a significant protective effect against MPP^+^-induced cytotoxicity, although only for the smallest concentration of MPP^+^ tested. These fiscalins could be important ROS scavengers and inhibit their production, therefore having a beneficial effect on the OS induced by the MPP^+^.

So far, the data obtained allowed us to suggest that stereochemistry also affected the protective profile of the tested compounds, which was verified by comparing the results obtained for fiscalins **1a**, **1b** and **1c** (Figure 1). In this case, it was observed that the *S* stereochemistry at C-4 (ring C, Figure 1) favoured the protective effects against MPP^+^-induced cytotoxicity after 24 and 48 h of cell treatment. By altering the stereochemistry from *S* to *R*, the protective capacity was lost, with no significant effects being observed for fiscalin **1c** (*R* stereochemistry at C-4) when compared with fiscalins **1a** and **1b** (*S* stereochemistry at C-4), which were shown to significantly protect differentiated SH-SY5Y cells against MPP^+^-induced cytotoxicity. In addition, the *R* stereochemistry in the C-1 position could have also led to the protective effects observed for both fiscalins **1b** and **2a**. Similar results were obtained for fiscalins **2a** and **2b**, since fiscalin **2a**, with *S* stereochemistry at C-4, presented a protective effect, and fiscalin **2b**, with *R* stereochemistry at C-4, had no protective effect observed. However, this trend was shown to be inverted by the insertion of the hydroxyl group in the anthranilic portion of the fiscalin scaffold (ring A, Figure 1), as observed for fiscalin **6**, with *R* stereochemistry at C-4, probably due to the known antioxidant activity of phenolic groups [76,77]. Surprisingly, the protective effect of fiscalins **2a** and **6** was only observed 48 h after exposure, and for the smallest MPP^+^ concentration tested (250 μM), revealing a late protective effect. Overall, the obtained results suggest that, amongst all fiscalin derivatives tested, fiscalin **1a** may have a promising neuroprotective effect, being potentially promising for the treatment of PD, or for the development of new drugs targeting this disease.

Iron has several important roles in healthy brain functions. However, when its homeostasis is disrupted and iron levels begin to increase, oxidative stress and cell death can be triggered. Iron may induce oxidative stress due to its central role in ROS generation, and because of it can also oxidise dopamine (DA) to a highly reactive quinone, which ultimately leads to cellular dysfunction or cell death [19,78]. Additionally, ferric iron represents an important pro-oxidant that triggers oxidative stress by inducing ROS generation, for example, in neuroblastoma cells [59,79]. The importance of iron-induced toxicity has been supported by the protective effects demonstrated by iron chelators observed in cell culture models used to study ND, such as the SH-SY5Y cell line [16,19,59,80,81]. Importantly, iron has already been implicated in the pathology of several ND, including AD and PD [16,18,82]. Moreover, metal ions, particularly iron, provide a promising pharmacological target for the treatment of ND [17,83].

For experimental studies, the iron (III)-NTA complex prevents the hydrolysis of iron (III) at physiological pH, and has been widely used as an iron supply for numerous biological studies, since NTA moderate affinity for iron makes FeNTA an excellent model for mimicking small ligands involved in metal-chaperone proteins [84]. Therefore, to evaluate the protective effect of fiscalins towards ferric iron-induced cytotoxicity, ferric nitrilotriacetic acid (FeNTA) was the selected agent. Iron’s cytotoxic effect was, therefore, evaluated in differentiated SH-SY5Y cells by the NR uptake assay, upon 24 and 48 h of exposure to FeNTA (0–1500 µM). The FeNTA concentration range was selected based in previous works, which demonstrated the FeNTA cytotoxic effect for concentrations of 250 µM, 1000 µM, and up to 2500 µM, and also in SH-SY5Y cells [59,85,86].

Fiscalin derivatives **1b**, **2b, 4** and **5** have demonstrated significant protective effects towards iron (III)-induced cytotoxicity. They may play a role in ROS scavenging or have iron-chelating properties that possibly explain these protective effects. Therefore, these fiscalins could have promising neuroprotective effects or be useful for the synthesis of new derivatives to be further used in ND pathogenesis. Nevertheless, fiscalin **4** was able to maintain its significant protective effect, by reducing FeNTA cytotoxicity towards differentiated SH-SY5Y cells, upon 24 as well as 48 h of exposure, being the only tested fiscalin that showed a potentially sustained protective effect against iron (III)-induced cytotoxicity. Therefore, the obtained results suggest that this fiscalin may have a neuroprotective effect against metal-induced neurotoxicity, and adding this effect to its inhibitory effect on P-gp, it could be potentially promising for the treatment or for the development of new drugs targeting ND. Despite this, some of the tested fiscalins significantly increased FeNTA’s cytotoxic effect towards differentiated SH-SY5Y cells, including fiscalin **2b**, which after 48 h lost its protective effect seen right after 24 h of exposure. Accordingly, the pro-oxidant behaviour of several compounds has been suggested over the years. For instance, compounds that have proven antioxidant properties have demonstrated to also have a pro-oxidant behaviour [87]. An example of that is the widely accepted antioxidant vitamin C, which can also become a pro-oxidant when combined with iron, by reducing Fe^3+^ to Fe^2+^, which consequently leads to hydroxyl radicals and hydrogen peroxide generation [87,88,89]. In addition, α-Tocopherol, which is another powerful antioxidant, in high concentrations can become a pro-oxidant due to its antioxidant mechanism. When α-Tocopherol reacts with a free radical it can become a radical itself, becoming highly reactive in the absence of sufficient antioxidant defences [87]. Moreover, myricetin, a flavonoid with antioxidant activity, has the ability to scavenge ROS as well as to chelate iron. However, it gains pro-oxidant properties due to the reduction of molecular oxygen and Fe^3+^ to Fe^2+^ [90]. Regarding fiscalins **2b**, **4** and **5**, their protective effect towards FeNTA-induced cytotoxicity could be mostly related to a direct effect on iron, given the lack of any protective effect against MPP^+^-induced cytotoxicity. Additionally, fiscalin **1b** could be interfering in the OS imbalance by scavenging ROS, despite any iron-chelating properties, due to the protective effect verified towards both FeNTA and MPP^+^ cytotoxicity.

Regarding our work, the analysis of the data obtained in the FeNTA experiments showed that overall, the insertion of a phenolic group substituent in the C-1 position led to an increased protective effect against iron-induced cytotoxicity for fiscalins **4** and **5.** In fact, the same stereochemistry (1*S*,4*S*) in the positions C-1 and C-4 (ring C, Figure 1) led to a long-term protection effect, verified for fiscalin **4**, which did not occur for the rest of the fiscalins, namely fiscalin **5**, with different stereochemistry (1*R*,4*S*). Furthermore, and attending to the stereochemistry, it was possible to assess that the presence of an isopropyl group at C-1, with a *R* configuration (fiscalin **1b**), and an isobutyl group at C-1, with a *S* configuration (fiscalin **2b**), were crucial to the protective effect against the ferric iron-induced cytotoxicity.

**Figure 6:**
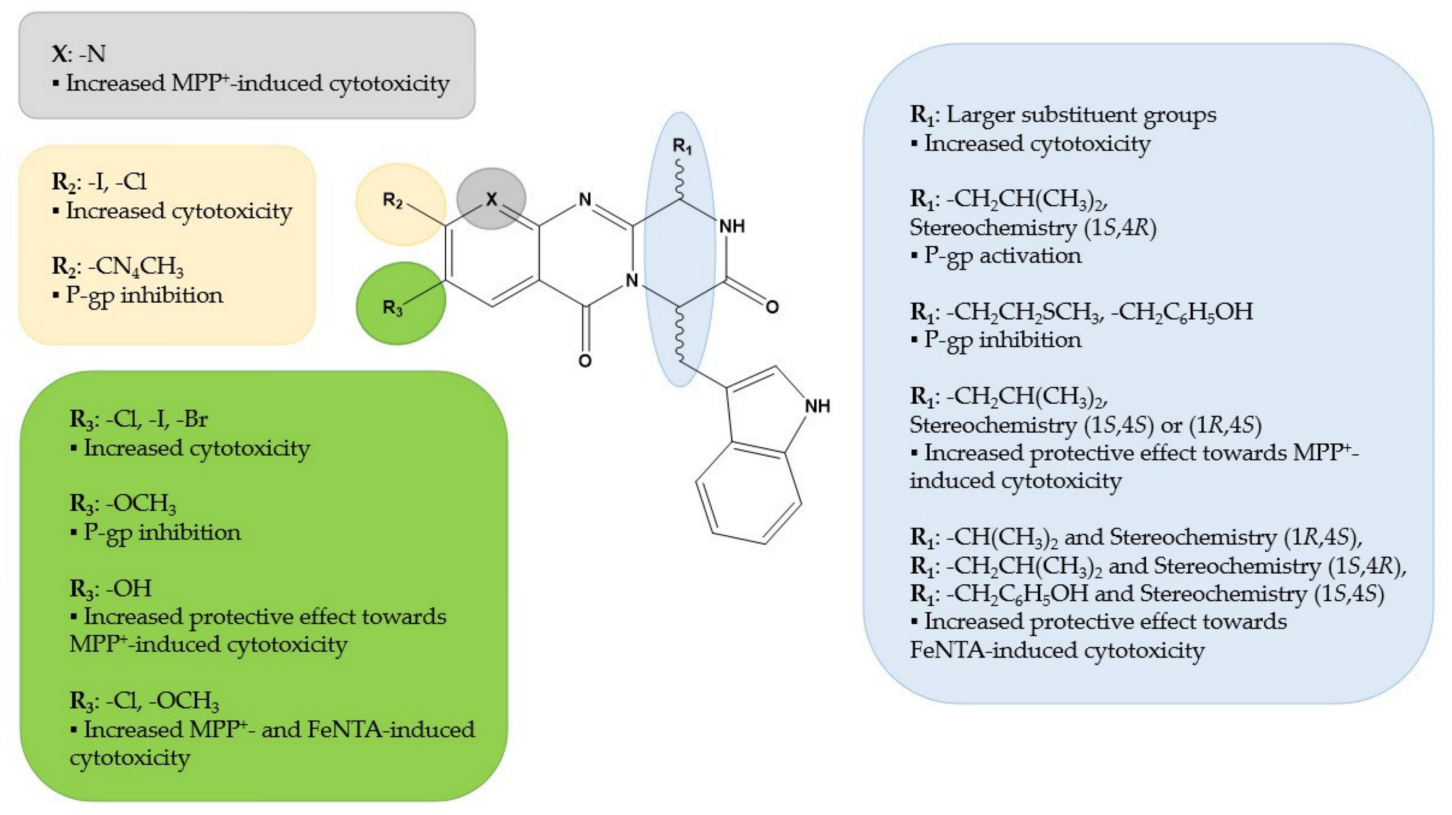
Structure–neuroprotective activity relationship for the fiscalin derivatives under investigation in the present study.

Lastly, taking the obtained results together, it is possible to extract some overall considerations concerning structure-protective activity amongst the library of fiscalin derivatives tested, as shown in Figure 6. First of all, the results obtained indicated that differences in stereochemistry were critical for the activity of these compounds. For instance, compounds **1a**, **1b** and **1c**, which are stereoisomers, presented different behaviours among the performed assays, highlighting the importance of the tridimensionality structure of compounds in their activity. Second, the substituent at position 1 seems to influence fiscalin activity, with short side chains, such as in fiscalins **1** and **2**, possibly prompting their neuroprotective effects. Moreover, adding an hydroxyl group on the aromatic substituent at C-1, such as in fiscalin **5**, was shown to be possibly beneficial for the protective effect, which is in accordance with the study on SH-SY5Y cells with cytotoxicity induced by rotenone [30]. Third, different substitutions in the aromatic ring of the anthranilic portion, besides halogens, did not seem to have helped the fiscalins’ protective activity.

**Figure 7:**
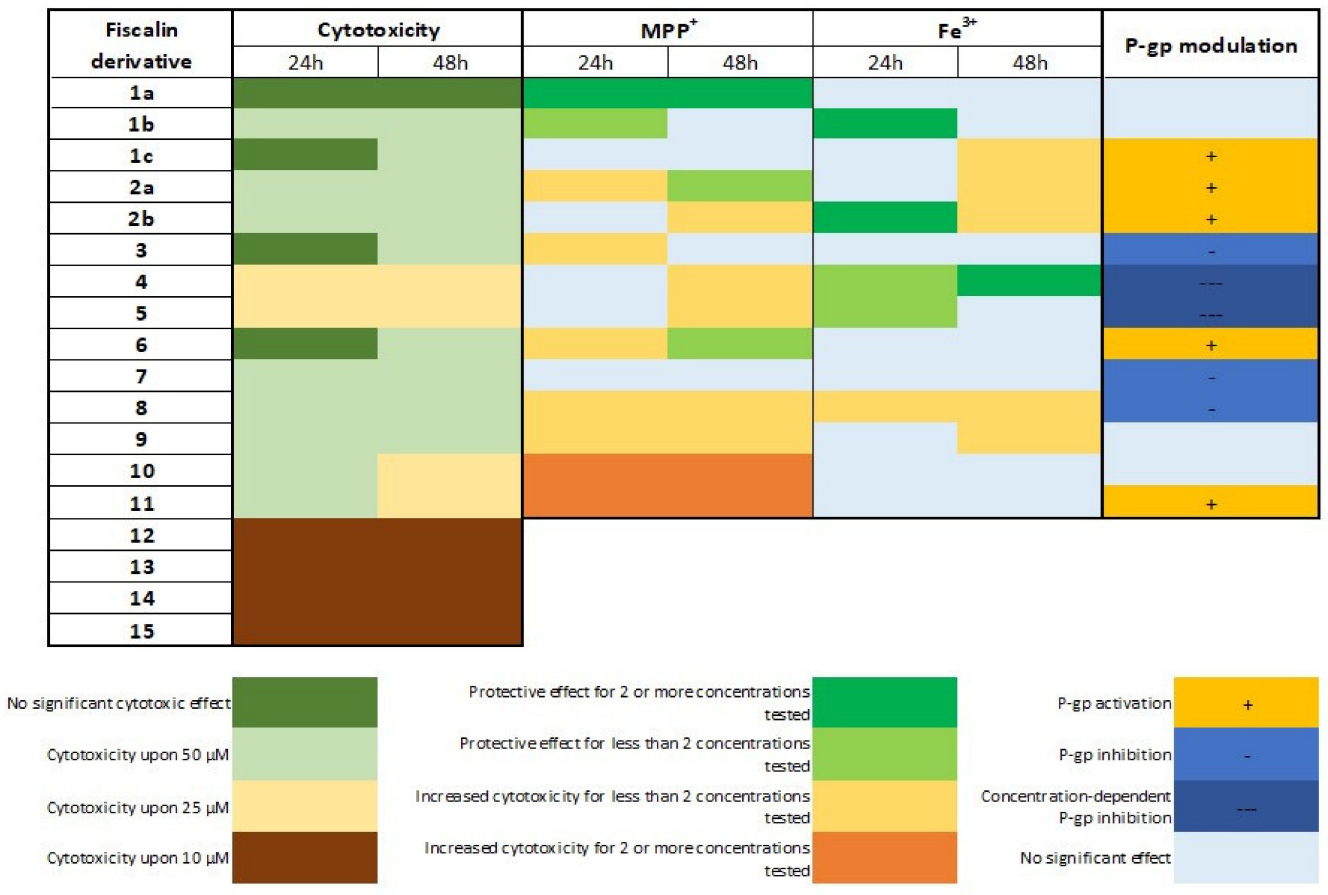
Overview of the results obtained for all the fiscalin derivatives (**1a**, **1b**, **1c**, **2a**, **2b**, **3**–**15**) under investigation in the present study.

Overall, it is important to highlight that amongst all the fiscalin derivatives tested in this work (Figure 6 and Figure 7), fiscalin **1a** has proven to be a promising compound for the treatment of ND such as PD, given its sustained protective effect on differentiated SH-SY5Y cells towards MPP^+^-induced cytotoxicity, verified for both timepoints tested; moreover, fiscalin **1b** could be particularly important in the treatment or prevention of ND, given the protective effect observed in both disease models used upon this work (MPP^+^ and FeNTA). Additionally, fiscalin **2b**, due to its protective effect towards iron-induced cytotoxicity and the P-gp activation capacity, could be promising for the Aβ peptides detoxification, and therefore, for the treatment or prevention of AD. Furthermore, fiscalins **4** and **5** also revealed important biological activities with particular benefit for the treatment and/or prevention of ND, as they accumulate a concentration-dependent P-gp inhibitory capacity alongside with a sustained protective effect against FeNTA-induced cytotoxicity, particularly for fiscalin **4**. Overall, although further studies are needed, from the obtained data it is possible to highlight that the fiscalin scaffold seems to be a promising new source of neuroprotective agents that may open new perspectives in the treatment of ND, such as AD and PD.

## 5. Conclusions

In the present work, some fiscalin derivatives have shown relevant biological activities regarding the modulation of the P-gp, as well as protective effects towards cytotoxicity-inducing agents (MPP^+^ and Iron (III)). Fiscalins **1c**, **2a**, **2b**, **6** and **11** caused an increase in P-gp transport activity, while fiscalins **3**, **4**, **5**, **7** and **8** caused significant P-gp inhibition, thus suggesting a promising source of new P-gp activators and inhibitors, respectively. Moreover, fiscalins **1a**, **1b**, **2a** and **6**, particularly fiscalins **1a** and **1b**, showed a significant protective effect against MPP^+^-induced cytotoxicity, and fiscalins **1b**, **2b**, **4** and **5** showed a protective effect against iron (III)-induced cytotoxicity.

Despite the important biological properties presented in this work, the mechanisms underlying the observed neuroprotective effects are yet unknown, persisting the need for more mechanistic and in vivo studies to further elucidate their mechanisms of action and their potential as new disease-modifying drugs. Indeed, future perspectives of the present work aim at the evaluation, in vivo, of an MPTP model of PD, of the pharmacokinetics and pharmacodynamics profiles of the most promising fiscalin derivatives, as well as the assessment of potential side effects. Furthermore, and knowing that ferroptosis is involved in the etiology of several ND apart from PD and AD (such as amyotrophic lateral sclerosis or Huntington’s disease), these derivatives may also in the future represent new therapeutic strategies to be tested in the scope of the treatment and/or prevention of such diseases.

In conclusion, fiscalin derivatives, although with limited studies available, seem to be promising neuroprotective agents. Noteworthy, the fiscalin scaffold may provide a widespread library of new chemical structures with potential neuroprotective properties that could be relevant for the treatment of ND, namely AD and PD, and targeting different pathophysiological mechanisms of such diseases.

## Supplementary Materials

The following supporting information can be downloaded at: https://www.mdpi.com/article/10.3390/pharmaceutics14071456/s1, Figure S1: Rhodamine 123 (0–10 µM) and zosuquidar (0–5 µM) cytotoxicity evaluated in differentiated SH-SY5Y cells by the resazurin reduction (a), neutral red uptake (b) and sulforhodamine B binding (c) assays, 24 h after exposure; Figure S2: Optimization of the experimental conditions (rhodamine concentration and time of incubation) for the evaluation of P-gp activity in differentiated SH-SY5Y cells; Figure S3: Fiscalin (0–50 µM) cytotoxicity towards differentiated SH-SY5Y cells, evaluated by the resazurin reduction assay, 24 and 48 h after exposure; Figure S4: Fiscalin (0–50 µM) cytotoxicity towards differentiated SH-SY5Y cells, evaluated by the sulforhodamine B binding assay, 24 and 48 h after exposure; Figure S5: Evaluation of the fiscalins’ (0–25 μM) potential for P-glycoprotein activation and inhibition. P-glycoprotein activity was evaluated by fluorescence spectroscopy in differentiated SH-SY5Y cells exposed to the tested fiscalins (0–25 μM) during the 90 min incubation period with the fluorescent substrate (10 μM RHO 123); Figure S6: MPP+ (0–1500 µM) cytotoxicity evaluated in differentiated SH-SY5Y cells, in the presence or absence of fiscalins 1c, 2b, 3–5 and 7–11 (10 and 25 µM), evaluated by the neutral red uptake assay, 24 h after exposure; Figure S7: MPP+ (0–1500 µM) cytotoxicity evaluated in differentiated SH-SY5Y cells, in the presence or absence of fiscalins 1c, 2b, 3–5 and 7–11 (10 and 25 µM), evaluated by the neutral red uptake assay, 48 h after exposure; Figure S8: FeNTA (0–1500 µM) cytotoxicity evaluated in differentiated SH-SY5Y cells, in the presence or absence of fiscalins (10 and 25 µM), evaluated by the neutral red uptake assay, 24 h after exposure; Figure S9: FeNTA (0–1500 µM) cytotoxicity evaluated in differentiated SH-SY5Y cells, in the presence or absence of fiscalins (10 and 25 µM), evaluated by the neutral red uptake assay, 48 h after exposure.
