# Supplementary material for: Fiscalin Derivatives as Potential Neuroprotective Agents
Source: Pharmaceutics. 2022 Jul 12;14(7):1456. doi: 10.3390/pharmaceutics14071456 (PMC9320635; doi:10.3390/pharmaceutics14071456)
Supplement: Supplementary file 1 [file pharmaceutics-14-01456-s001.zip › pharmaceutics-1744625-supplementary.pdf]

pharmaceutics

MDPI

Article

Fiscalin Derivatives as Potential Neuroprotective Agents

Sandra Barreiro [1,2,*], Bárbara Silva [1,2], Solida Long [3,4], Madalena Pinto [4,5], Fernando Remião [1,2], Emília Sousa [4,5] and Renata Silva [1,2,*]

[1] Associate Laboratory i4HB - Institute for Health and Bioeconomy, Faculty of Pharmacy, University of Porto, 4050-313 Porto, Portugal; sandrafcbarreiro@gmail.com (S.B.); barbarapolerisilva@gmail.com (B.S.); remiao@ff.up.pt (F.R.); rsilva@ff.up.pt (R.S.)

[2] UCIBIO – Applied Molecular Biosciences Unit, Requimte, Laboratory of Toxicology, Department of Biological Sciences, Faculty of Pharmacy, University of Porto, 4050-313 Porto, Portugal;

[3] Department of Bioengineering, Royal University of Phnom Penh, Russian confederation Blvd. 12156, Phnom Penh, Cambodia; solidachhann@gmail.com

[4] CIIMAR - Centro Interdisciplinar de Investigação Marinha e Ambiental, Terminal de Cruzeiros do Porto de Leixões, 4450-208 Matosinhos, Portugal; madalenakijjoa@gmail.com (M.P.); esousa@ff.up.pt (E.S.)

[5] Laboratory of Organic and Pharmaceutical Chemistry, Department of Chemical Sciences, Faculty of Pharmacy, University of Porto, 4050-313 Porto, Portugal

* Correspondence: S.B.: sandrafcbarreiro@gmail.com; and R.S.: rsilva@ff.up.pt

Supplementary Material

A. Zosuquidar and RHO 123 cytotoxicity and optimization of the experimental conditions

SH-SY5Y cells were seeded at a density of 25,000 cells/cm^2 into 96-well plates, and were then submitted to a RA and TPA differentiation protocol. Six days after seeding, differentiated SH-SY5Y cells were exposed to ZOS (0 – 5 μM) and RHO 123 (0 – 10 μM) prepared in fresh cell culture medium. Subsequently, the plates were incubated, at 37 °C, in a humidified 5% CO_2 – 95% air atmosphere, for 24 hours. Following the incubation period, the cytotoxicity of both ZOS and RHO 123 was evaluated by the NR uptake, REZ reduction and SRB binding assays, as described in Section 2. Three independent experiments were performed, in triplicate.

Regarding RHO 123 (0 – 10 μM) cytotoxicity, no significant cytotoxic effect towards differentiated SH-SY5Y cells was observed for any of the tested concentrations when compared to the control cells (0 μM), and as evaluated by the NR uptake, REZ reduction and SRB binding assays, 24 hours after exposure to the P-gp substrate (Figure S1). Likewise, in the NR uptake, REZ reduction and SRB binding assays, no significant cytotoxic effect was observed 24 hours after exposure of differentiated SH-SY5Y cells to ZOS (0 – 5 μM), at any the tested concentrations and when compared to the control cells (0 μM) (Figure S1). Therefore, based on the obtained results, and since no significant cytotoxicity was observed towards differentiated SH-SY5Y cells, P-gp transport activity can be safely assessed using RHO 123 and ZOS at concentrations up to 10 μM and to 5 μM, respectively.

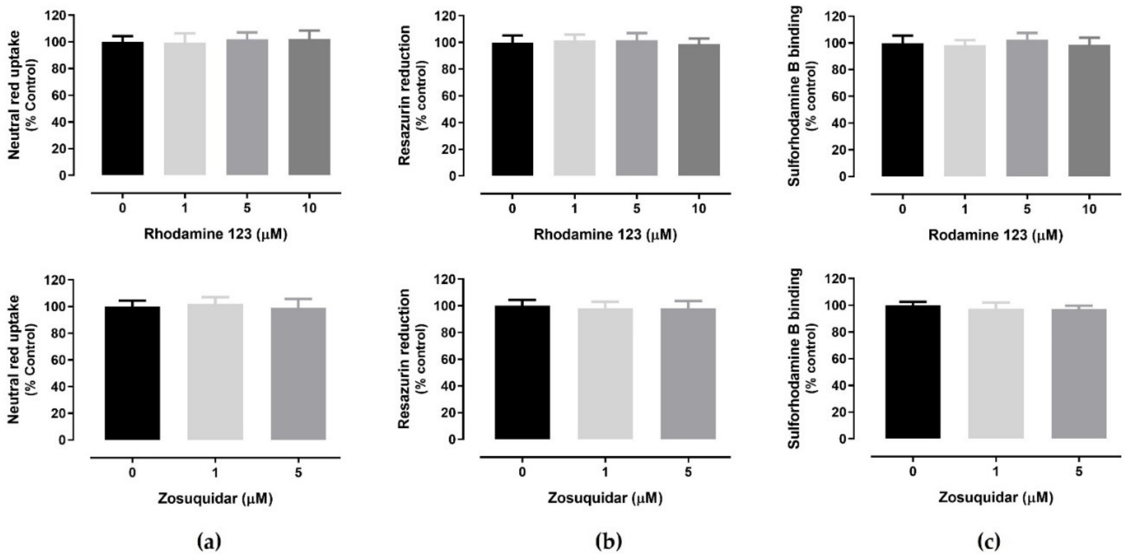

Figure S1. Rhodamine 123 (0 – 10 μM) and zosuquidar (0 – 5 μM) cytotoxicity evaluated in differentiated SH-SY5Y cells by the resazurin reduction (a), neutral red uptake (b) and sulforhodamine B binding (c) assays, 24 hours after exposure. Results are presented as mean ± SD from three independent experiments, performed in triplicate. Statistical comparisons were made using the parametric method of one-way ANOVA, followed by Dunnett's multiple comparisons test. In all cases, p values < 0.05 were considered significant.

Additionally, the protocol was optimised concerning the RHO 123 and ZOS concentrations to be used, as well as the duration of the incubation with the P-gp fluorescent substrate. For that purpose, SH-SY5Y cells were seeded in 24-well plates at a density of 25,000 cells/cm^2, and further differentiated through the RA and TPA differentiation protocol, as described in Section 2. Six days after seeding, differentiated SH-SY5Y cells were incubated with noncytotoxic concentrations of RHO 123 (0–10 μM), with or without preincubation with the P-gp inhibitor, ZOS (5 μM), for 30 minutes. The accumulation of the P-gp fluorescent substrate was then evaluated after 90 and 120 minutes of incubation. After the incubation period, the cells were washed twice with HBSS (+/+) and lysed with 0.1% Triton™ X100, for 30 min, at room temperature and in the absence of light. After lysis, the intracellular RHO 123 fluorescence was measured in a multiwell plate reader (PowerWave-X, BioTek Instruments, Vermont, USA) at excitation/emission wavelengths of 485/528 nm, respectively, and expressed as fluorescence intensity (FI). Three independent experiments were performed, in triplicate.

Accordingly with the obtained results, and as expected, the RHO 123 intracellular accumulation under ZOS-mediated P-gp inhibition was significantly higher for the 10 μM RHO 123 concentration than for the 5 μM concentration when compared to the control cells, which were exposed to RHO 123 in the absence of ZOS (Figure S2). Furthermore, this higher intracellular RHO 123 accumulation detected for the highest tested concentration was observed for both timepoints (90 and 120 minutes). Furthermore, for the 5 μM RHO 123 concentration, and upon 120 minutes of incubation, the accumulation of the P-gp fluorescent substrate in the presence of the P-gp inhibitor was significantly higher when compared to the 90 minute timepoint (RHO 123 intracellular accumulation significantly increased to 299.9% and 388.5% after 90 and 120 minutes of incubation with 5 μM RHO 123 under ZOS-mediated P-gp inhibition, respectively, and when compared with cells incubated with the P-gp fluorescent substrate in the absence of ZOS) (Figure S2). However, concerning the 10 μM RHO 123 concentration, the RHO 123 accumulation was similar for both timepoints, as shown in Figure S2 (RHO 123 intracellular accumulation significantly increased to 435.6% and 439.2% after 90 and 120 minutes of

incubation with 10 μM RHO 123 in the presence of ZOS, respectively, when compared with cells incubated with the P-gp fluorescent substrate in the absence of the P-gp inhibitor). Given these results, for the evaluation of the fiscalin derivatives' effects on P-gp transport activity, the 10 μM RHO 123 concentration was selected, as it was the concentration leading to a significatively higher RHO 123 intracellular accumulation in the differentiated SH-SY5Y cells. Furthermore, given the RHO 123 concentration selected, and since no significant differences were observed for both timepoints, the 90 minute accumulation period was selected for the subsequent experiments.

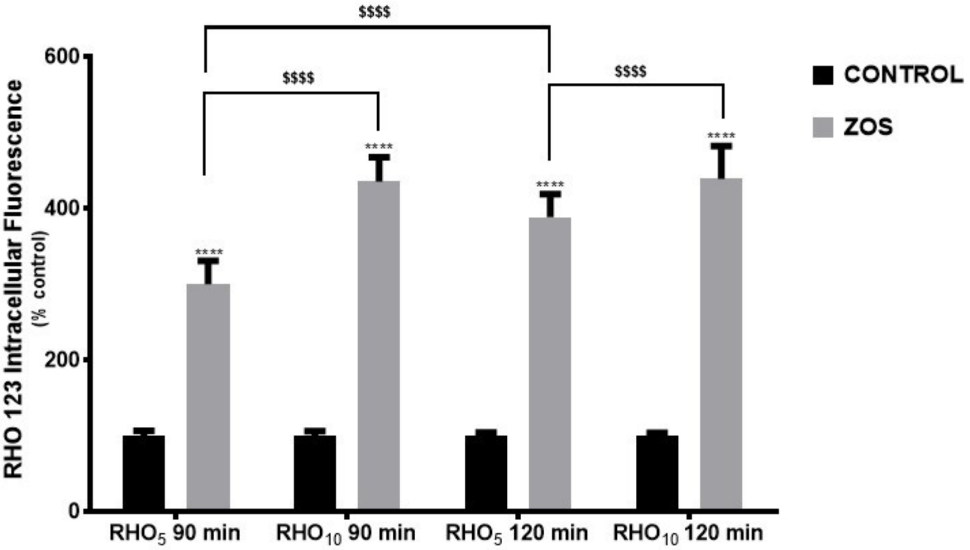

Figure S2. Optimization of the experimental conditions (rhodamine concentration and time of incubation) for the evaluation of P-gp activity in differentiated SH-SY5Y cells. Results are presented as mean ± SD from three independent experiments, performed in triplicate. Statistical comparisons were made using two-way ANOVA, followed by Sidak's multiple comparisons test [for each condition, for comparisons between control and ZOS-treated cells (****$p < 0.0001$)] or by Tukey's multiple comparisons test [for comparison between conditions (RHO concentration and time of incubation) in ZOS-treated cells ($$$$ $p < 0.0001$)]. In all cases, p values < 0.05 were considered significant.

B. Fiscalins' cytotoxicity – REZ reduction and SRB binding assays

In the REZ reduction assay (Figure S3), no significant effects were detected 24 hours after exposure to fiscalins **1a**, **1b**, **1c**, **2a**, **2b**, **3**, **4**, **5**, **6** and **8**, at any of the tested concentration. However, a significant decrease in REZ was detected 48 hours after exposure to the highest concentration of fiscalins **1a**, **1b**, **2a**, **2b**, **3, 6** and **8.** For fiscalins **1c, 4** and **5** the significant REZ reduction, although small, was detected 48 hours after exposure to 25 and 50 μM. For fiscalin **10**, a significant decrease in REZ reduction was observed 24 hours after exposure to the highest tested concentration (50 μM), an effect intensified at the 48-hour timepoint, with significant effects also being observed at 25 μM. On the other hand, for fiscalin **11** a significant effect was detected at both timepoints, but only at the highest tested concentration (50 μM). For fiscalins **7** and **9**, no significant cytotoxic effects were detected at any tested concentration or exposure time. On the opposite, the most cytotoxic compounds were fiscalins **12, 13, 14** and **15**, with significant decreases in REZ reduction being detected for the two highest concentrations (25 and 50 μM) at both timepoints tested. Moreover, for fiscalins **13** and **14** a significant decrease in REZ reduction was even observed at 10 μM, 48 hours after exposure. Similar results were obtained for the SRB binding assay (Figure S4).

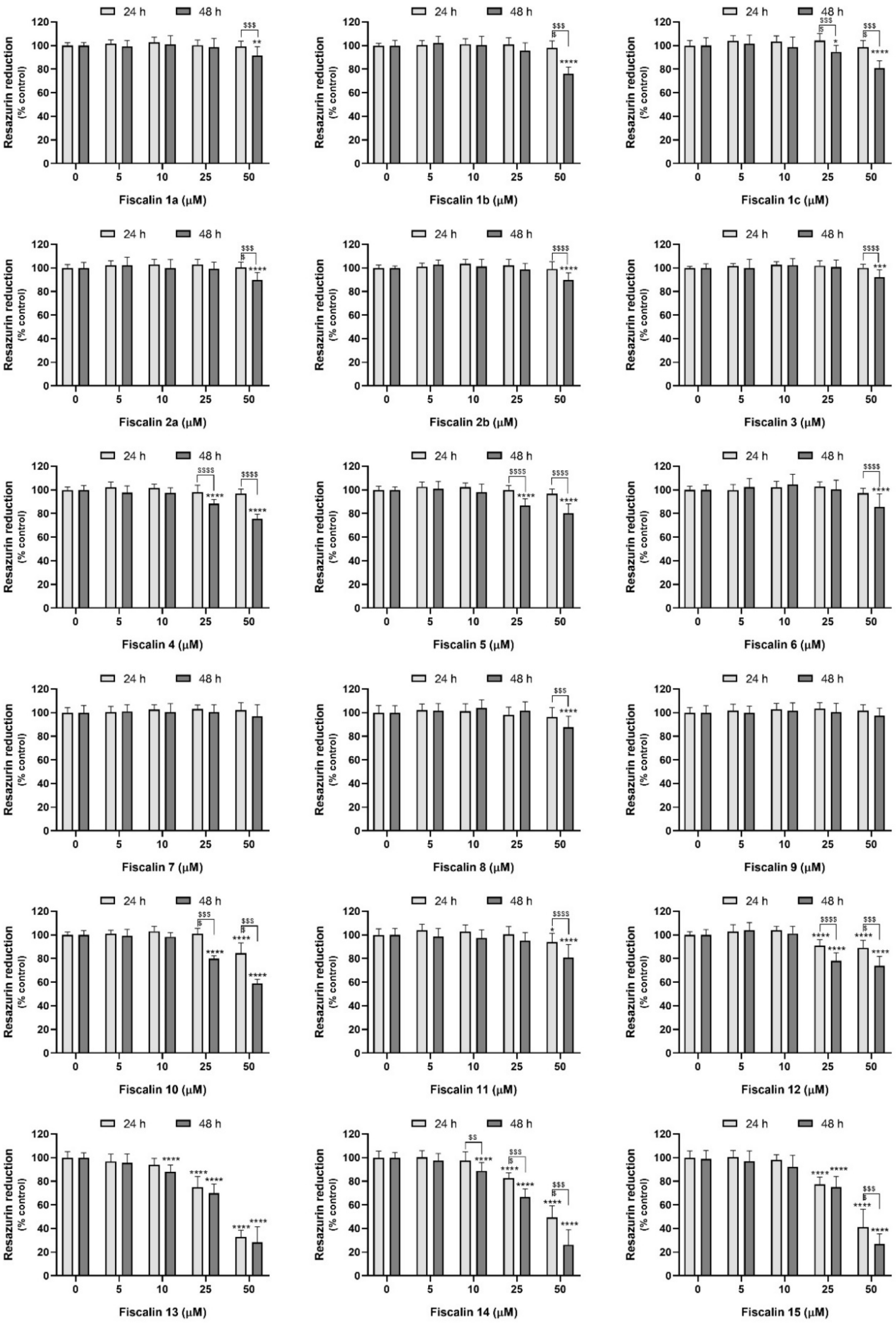

Figure S3. Fiscalins (0 – 50 μM) cytotoxicity towards differentiated SH-SY5Y cells, evaluated by the resazurin reduction assay, 24 and 48 hours after exposure. Results are presented as mean ± SD from at least four independent experiments, performed in triplicate. Statistical comparisons were made using two-way ANOVA, followed by Tukey's multiple comparisons test (at each timepoint, for comparisons between concentrations) or by Sidak's multiple comparisons test (for each concentration, for comparisons between timepoints). In all cases, p values < 0.05 were considered significant [$^{*}p < 0.05$; $^{***}p < 0.001$; $^{****}p < 0.0001$ *versus* control (0 μM), at each timepoint; $^{\$\$}p < 0.01$; $^{\$\$\$}p < 0.001$; $^{\$\$\$\$}p < 0.0001$, 24 hours *versus* 48 hours, at each concentration].

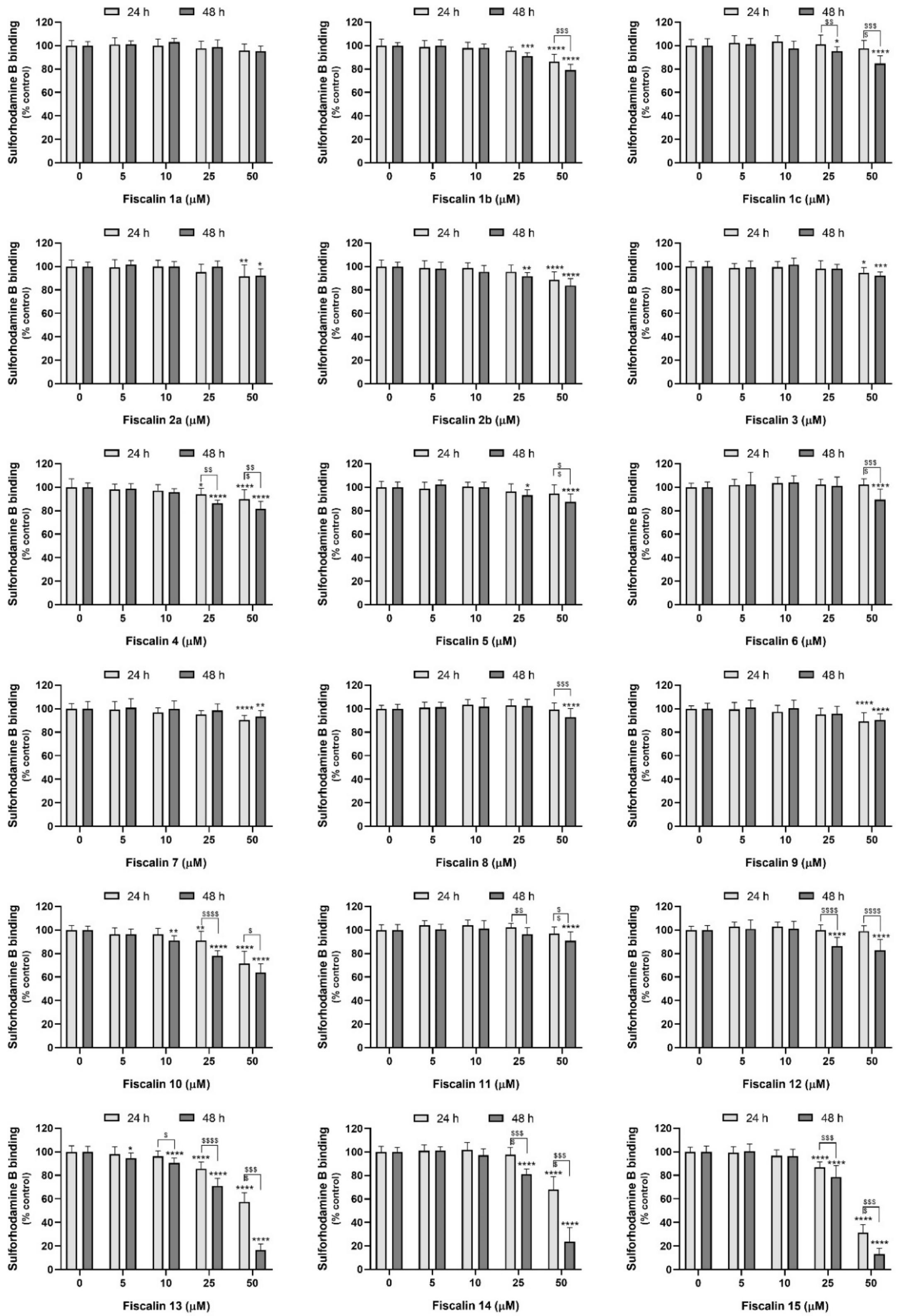

Figure S4. Fiscalins (0 – 50 μM) cytotoxicity towards differentiated SH-SY5Y cells, evaluated by the sulforhodamine B binding assay, 24 and 48 hours after exposure. Results are presented as mean ± SD from at least four independent experiments, performed in triplicate. Statistical comparisons were made using two-way ANOVA, followed by Tukey's multiple comparisons test (at each timepoint, for comparisons between concentrations) or by Sidak's multiple comparisons test (for each concentration, for comparisons between timepoints). In all cases, p values < 0.05 were considered significant [$^{*}p < 0.05$; $^{**}p < 0.01$; $^{***}p < 0.001$; $^{****}p < 0.0001$ *versus* control (0 μM), at each timepoint; $^{\$}p < 0.05$; $^{\$\$}p < 0.01$; $^{\$\$\$}p < 0.001$; $^{\$\$\$\$}p < 0.0001$ 24 hours versus 48 hours, at each concentration].

C. Fiscalins' modulatory effect on P-glycoprotein

Concerning the evaluation of the P-gp activity in the presence of the fiscalins **1a**, **1b**, **1c**, **2a**, **2b**, **3 – 11** (0 – 25 μM) in the presence and absence of ZOS (5 μM), through the RHO 123 accumulation assay, no significant effect on P-gp activity was observed for fiscalins **1a**, **1b**, **9** and **10**, at any of the tested concentrations, and when compared to control cells (0 μM), as shown in Figure S5.

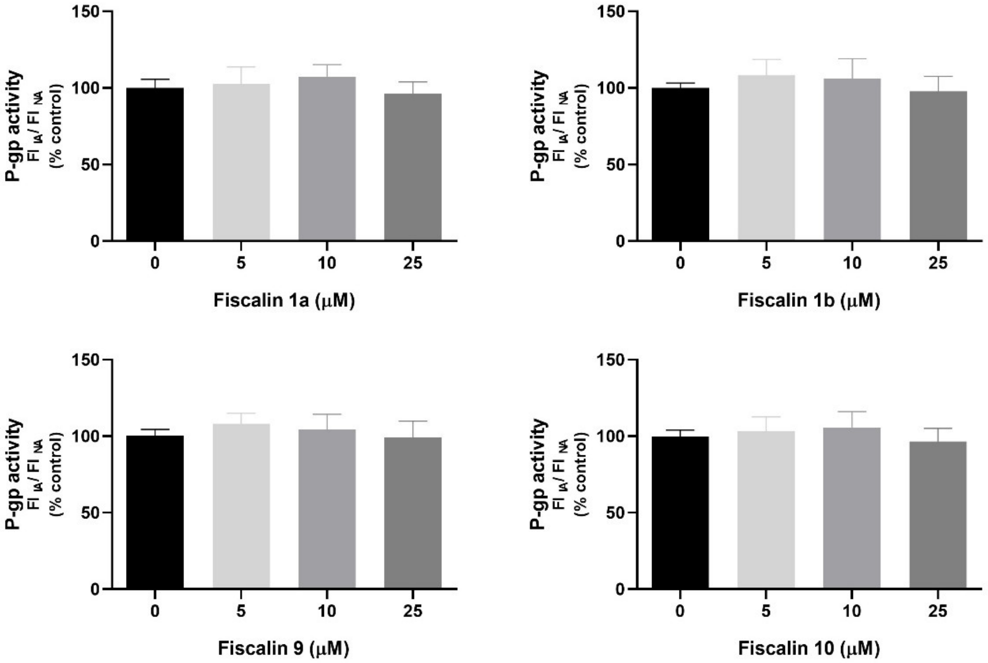

Figure S5. Evaluation of the fiscalins (0 – 25 μM) potential for P-glycoprotein activation and inhibition. P-glycoprotein activity was evaluated by fluorescence spectroscopy in differentiated SH-SY5Y cells exposed to the tested fiscalins (0 – 25 μM) during the 90-minute incubation period with the fluorescent substrate (10 μM RHO 123). Results are presented as mean ± SD from four independent experiments, performed in triplicate. Statistical comparisons were made using the parametric method of one-way ANOVA, followed by Dunnett's multiple comparisons test [*$p < 0.05$; **$p < 0.01$; ***$p < 0.001$; ****$p < 0.0001$ *vs* control (0 μM)].

D. Evaluation of fiscalins' protective effects

a) MPP⁺-induced cytotoxicity

Concerning the protective effect of the tested fiscalin derivatives towards MPP^+-induced cytotoxicity, fiscalins **1c**, **2b**, **4**, **5** and **7** did not affect MPP^+-induced cytotoxicity at any concentration (Figure S6). Conversely, fiscalins **3**, **8**, **9**, **10** and **11** were found to increase MPP^+-induced cytotoxicity towards differentiated SH-SY5Y cells (Figure S6).

For compound **3**, and 24 hours after exposure, the MPP^+ cytotoxic effect was increased for its highest concentration tested (1500 μM) in the presence of compound **3** at the 25 μM concentration, as shown by the significant decrease in the NR uptake when compared to MPP^+ alone (64.4% for 1500 μM MPP^+ in the presence of 25 μM compound **3**, when compared to 75.3% observed for 1500 μM MPP^+ alone).

A significant reduction in the NR uptake was also observed after exposure to 1500 μM of MPP^+ in the presence of fiscalin **8** (Figure S6), at 10 μM (64.7%), and also for concentrations equal or above 1000 μM of MPP^+, in the presence of fiscalin **8** at the noncytotoxic concentration of 25 μM, demonstrating the ability of this compound for worsening MPP^+-induced cytotoxicity (NR uptake significantly decreased to 69.2% and 58.4% after

24 hours of exposure to 1000 and 1500 µM MPP⁺ in the presence of 25 µM fiscalin **8**, when compared to 79.0% and 75.5% observed for MPP⁺ alone).

A significant reduction in the NR uptake was also observed for fiscalin **9** (Figure S6), for both tested concentrations, 24 hours after exposure (72.2% and 62.7% 24 hours after exposure to 1000 and 1500 µM of MPP⁺ in the presence of 10 µM of fiscalin **9**; and 78.0%, 67.7% and 56.6%, 24 hours after exposure to 500, 1000 and 1500 µM MPP⁺ in the presence of 25 µM fiscalin **9**, and when compared to 85.7%, 79.3% and 75.4%, 24 hours after exposure to 500, 1000 and 1500 µM of MPP⁺ alone).

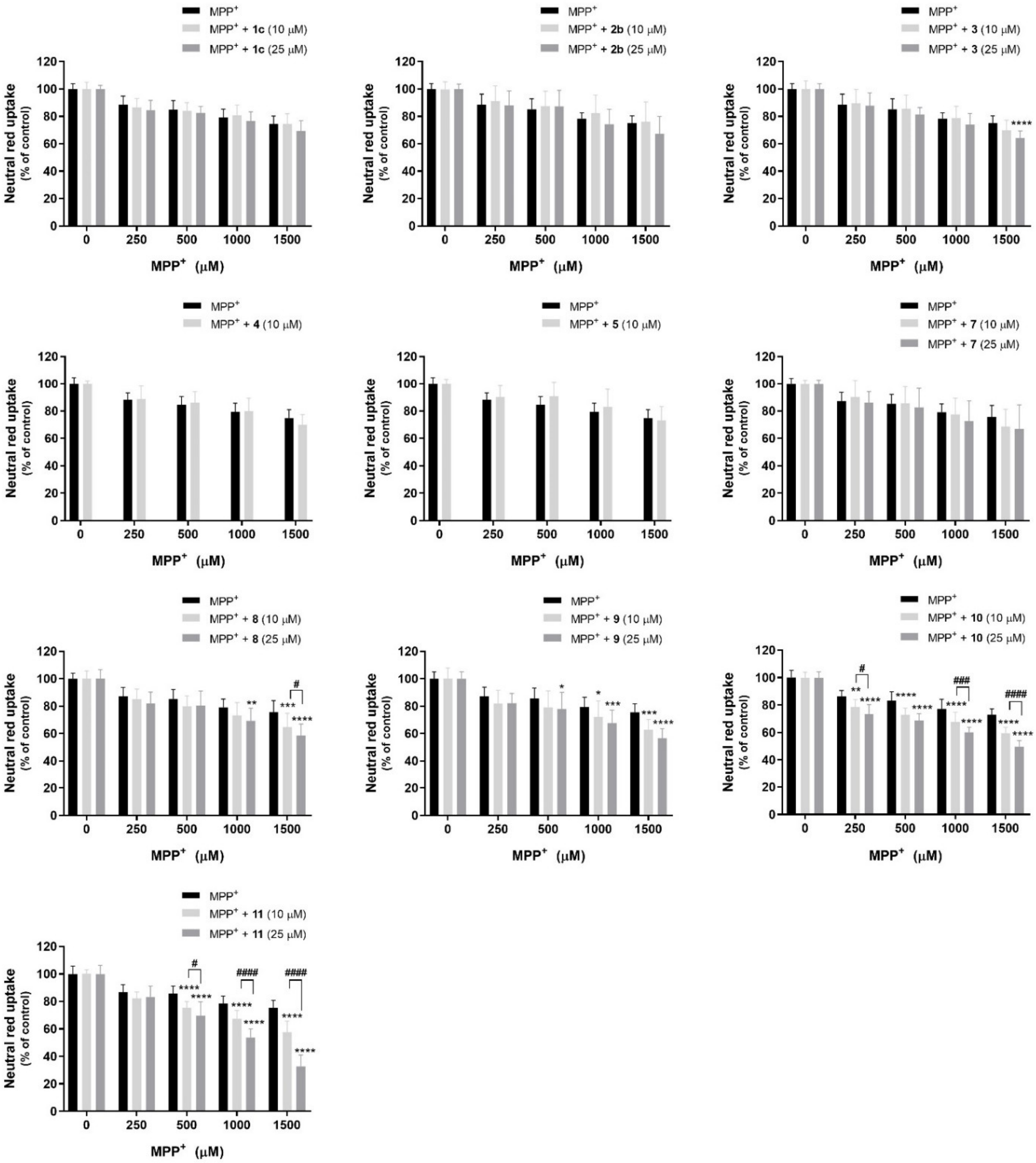

Figure S6. MPP⁺ (0 – 1500 µM) cytotoxicity evaluated in differentiated SH-SY5Y cells, in the presence or absence of fiscalins 1c, 2b, 3–5 and 7–11 (10 and 25 µM), evaluated by the neutral red uptake assay, 24 hours after exposure. Results are presented as mean ± SD from 4 independent experiments, performed in triplicate. Statistical comparisons were made using two-way ANOVA, followed by Holm–Šídák's multiple comparisons test. In all cases, p values < 0.05 were considered significant [*$p < 0.05$; **$p < 0.001$; ***$p < 0.001$; ****$p < 0.0001$ *versus* MPP⁺ alone].

Moreover, as shown in Figure S6, in the presence of fiscalin **10** (10 and 25 μM), a significant decrease in the NR uptake was observed for all the tested MPP+ concentrations (250–1500 μM) when compared to MPP+ alone (NR significantly decreased to 78.7%, 72.8%, 67.7% and 59.6%, 24 hours after exposure to 250, 500, 1000 and 1500 μM MPP+ in the presence of 10 μM compound **10**; and to 73.5%, 68.8%, 60.2% and 49.7%, 24 hours after exposure to 250, 500, 1000 and 1500 μM MPP+ in the presence of 25 μM compound **10**, and when compared to 86.2%, 83.1%, 77.3% and 73.0% for MPP+ alone).

Likewise, in the presence of compound **11** (10 and 25 μM), a significant decrease in the NR uptake was observed for concentrations equal or above 500 μM of MPP+ (NR significantly decreased to 75.2%, 67.4% and 57.5%, 24 hours after exposure to 500, 1000 and 1500 μM MPP+ in the presence of 10 μM compound **11**; and to 69.5%, 53.7% and 32.7%, 24 hours after exposure to 500, 1000 and 1500 μM MPP+ in the presence of 25 μM compound **11**, when compared to 85.6%, 78.6% and 75.3% observed for MPP+ alone).

In the NR uptake assay, 48 h after exposure, fiscalins **1c**, **3** and **7** did not affect MPP+-induced cytotoxicity at any concentration. Conversely, fiscalins **2b**, **4**, **5**, **8**, **9**, **10** and **11** were found to increase MPP+-induced cytotoxicity towards differentiated SH-SY5Y.

For both 10 and 25 μM of fiscalins **2b, 8** and **9** (Figure S7), a significant decrease in the NR uptake was observed for concentrations equal or above 1000 μM of MPP+, and when compared to MPP+ alone (NR uptake significantly decreased to 62.9% and 50.6%, and to 62.1% and 45.9%, 48 hours after exposure to 1000 and 1500 μM of MPP+ in the presence of 10 μM and 25 μM of fiscalin **2b**, respectively, when compared to 68.4% and 58.5% observed for MPP+ alone; NR uptake significantly decreased to 56.1% and 41.1%, and to 54.5% and 38.2%, 48 hours after exposure to 1000 and 1500 μM of MPP+ in the presence of 10 μM and 25 μM of fiscalin **8**, respectively, when compared to 64.7% and 56.1% observed for MPP+ alone; and NR uptake significantly decreased to 56.4% and 41.2%, and to 54.7% and 40.2%, 48 hours after exposure to 1000 and 1500 μM of MPP+ in the presence of 10 μM and 25 μM of fiscalin **9**, respectively, when compared to 65.4% and 53.4% observed for MPP+ alone).

A significant increase in MPP+-induced cytotoxicity was also observed in the presence of fiscalin **4** (10 μM) and fiscalin **5** (10 μM) for concentrations equal to or above 500 μM of MPP+. Likewise, in the presence of fiscalins **10** and **11** (10 μM), a significant decrease in the NR uptake was observed for all the tested MPP+ concentrations (250–1500 μM), and when compared to MPP+ alone, as shown in Figure S7. Moreover, MPP+-induced cytotoxicity was significantly higher in the presence of the fiscalins **10** and **11**, upon 48 hours exposure, also being higher than what was observed for the 24-hour timepoint (NR uptake significantly decreased to 70.9%, 64.7%, 55.4% and 44.4%, 48 hours after exposure to 250, 500, 1000 and 1500 μM MPP+ in the presence of 10 μM of fiscalin **10;** to 65.0%, 56.5%, 46.0% and 34.0%, 48 hours after exposure to 250, 500, 1000 and 1500 μM MPP+ in the presence of 10 μM of fiscalin **11**, when compared to 76.3%, 70.1%, 62.5% and 55.5% observed for MPP+ alone).

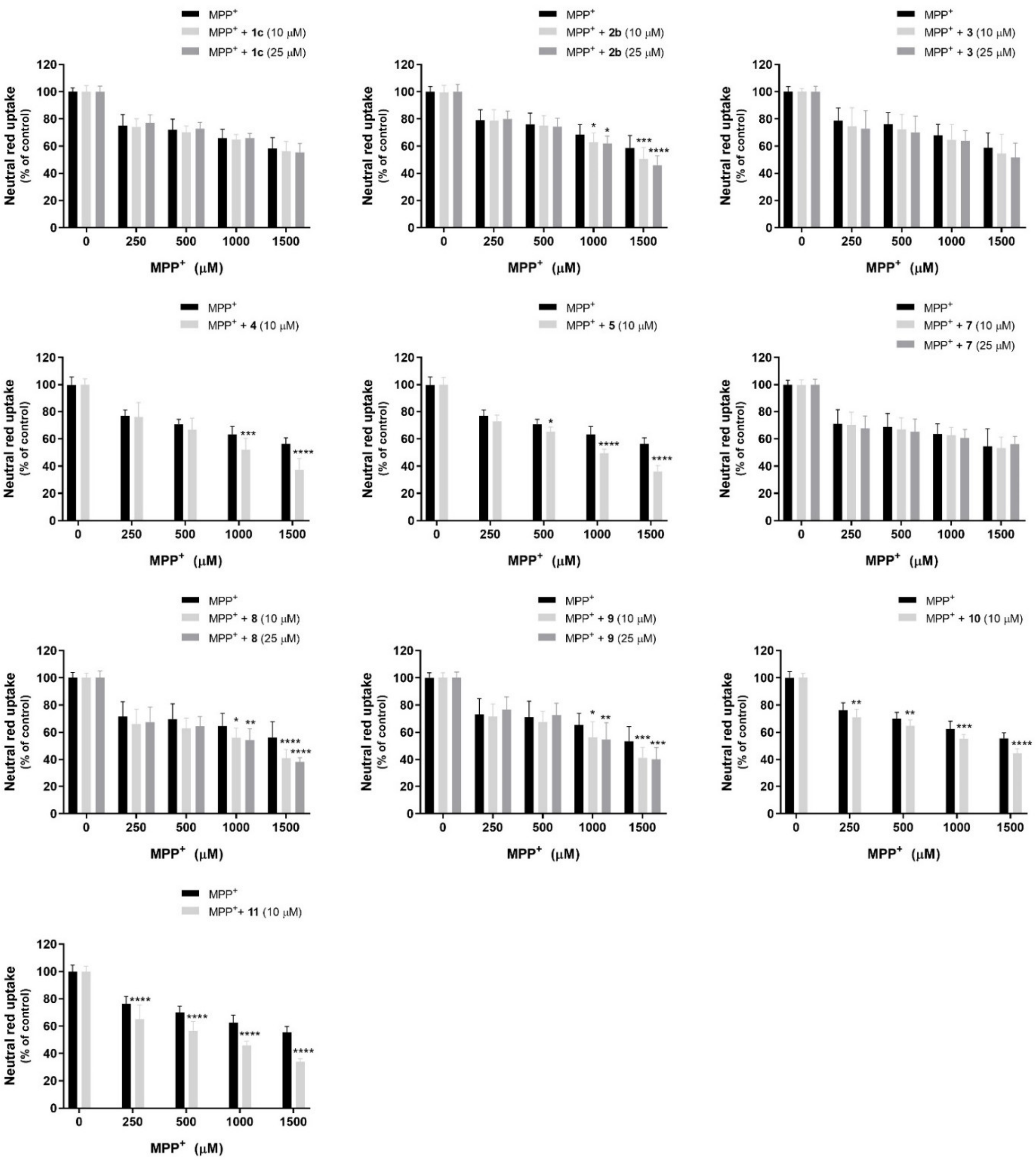

Figure S7. MPP^+ (0 – 1500 μM) cytotoxicity evaluated in differentiated SH-SY5Y cells, in the presence or absence of fiscalins 1c, 2b, 3-5 and 7-11 (10 and 25 μM), evaluated by the neutral red uptake assay, 48 hours after exposure. Results are presented as mean ± SD from 4 independent experiments, performed in triplicate. Statistical comparisons were made using two-way ANOVA, followed by Holm–Šídák's multiple comparisons test. In all cases, p values < 0.05 were considered significant [*$p < 0.05$; **$p < 0.001$; ***$p < 0.001$; ****$p < 0.0001$ *versus* MPP^+ alone].

b) FeNTA-induced cytotoxicity

Concerning the protective effect of the tested fiscalin derivatives towards FeNTA-induced cytotoxicity, and 24 hours after exposure, fiscalins **1a**, **1c**, **2a**, **3**, **6**, **7**, **9**, **10** and **11** did not affect iron (III)-induced cytotoxicity at any concentration tested (Figure S8). However, fiscalin **8** was found to increase FeNTA-induced cytotoxicity towards differentiated

SH-SY5Y cells. In the presence of fiscalin **8** (Figure S8), at 10 and 25 μM, a small but significant decrease in the NR uptake was observed for 500 and 1000 μM of FeNTA (NR uptake significantly decreased to 83.6% and 69.7%, in the presence of 10 μM of fiscalin **8**; and to 84.5% and 70.7%, in the presence of 25 μM of fiscalin **8**, 24 hours after exposure to 500 and 1000 μM of FeNTA, respectively, and when compared to 89.8% and 77.9% for 500 and 1000 μM of FeNTA alone).

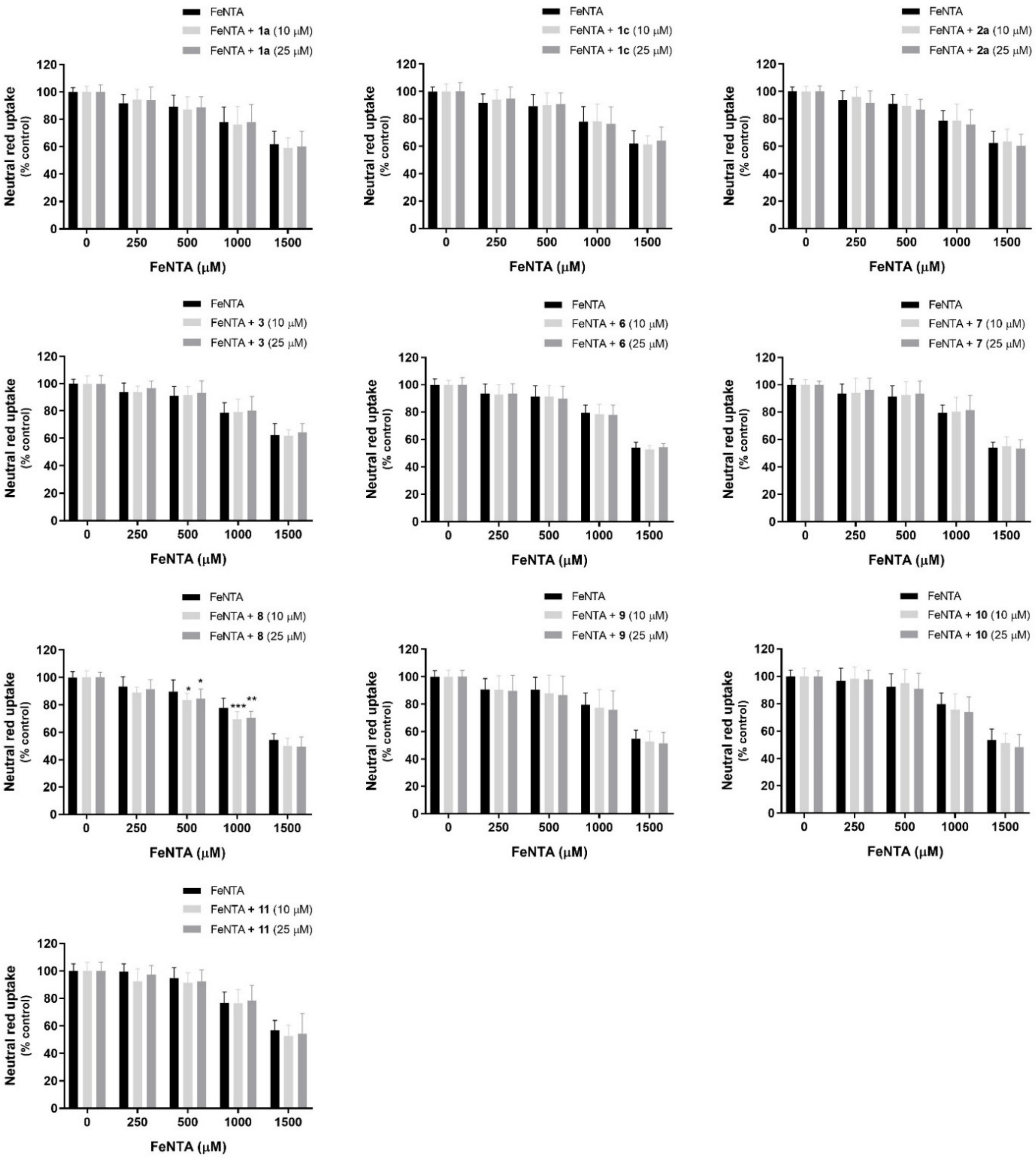

Figure S8. FeNTA (0 – 1500 μM) cytotoxicity evaluated in differentiated SH-SY5Y cells, in the presence or absence of fiscalins (10 and 25 μM), evaluated by the neutral red uptake assay, 24 hours after exposure. Results are presented as mean ± SD from 4 independent experiments, performed in triplicate. Statistical comparisons were made using two-way ANOVA, followed by Holm–Šídák's multiple comparisons test. In all cases, p values < 0.05 were considered significant [*$p < 0.05$; **$p < 0.001$; ***$p < 0.001$; ****$p < 0.0001$ *versus* FeNTA alone].

Additionally, 48 hours after exposure, fiscalins **1a**, **3**, **6**, **10** and **11** did not affect iron (III)-induced cytotoxicity at any concentration tested (Figure S9). Conversely, fiscalins **1c**, **2a**, **7**, **8** and **9** were found to increase FeNTA-induced cytotoxicity towards differentiated SH-SY5Y cells (Figure S9).

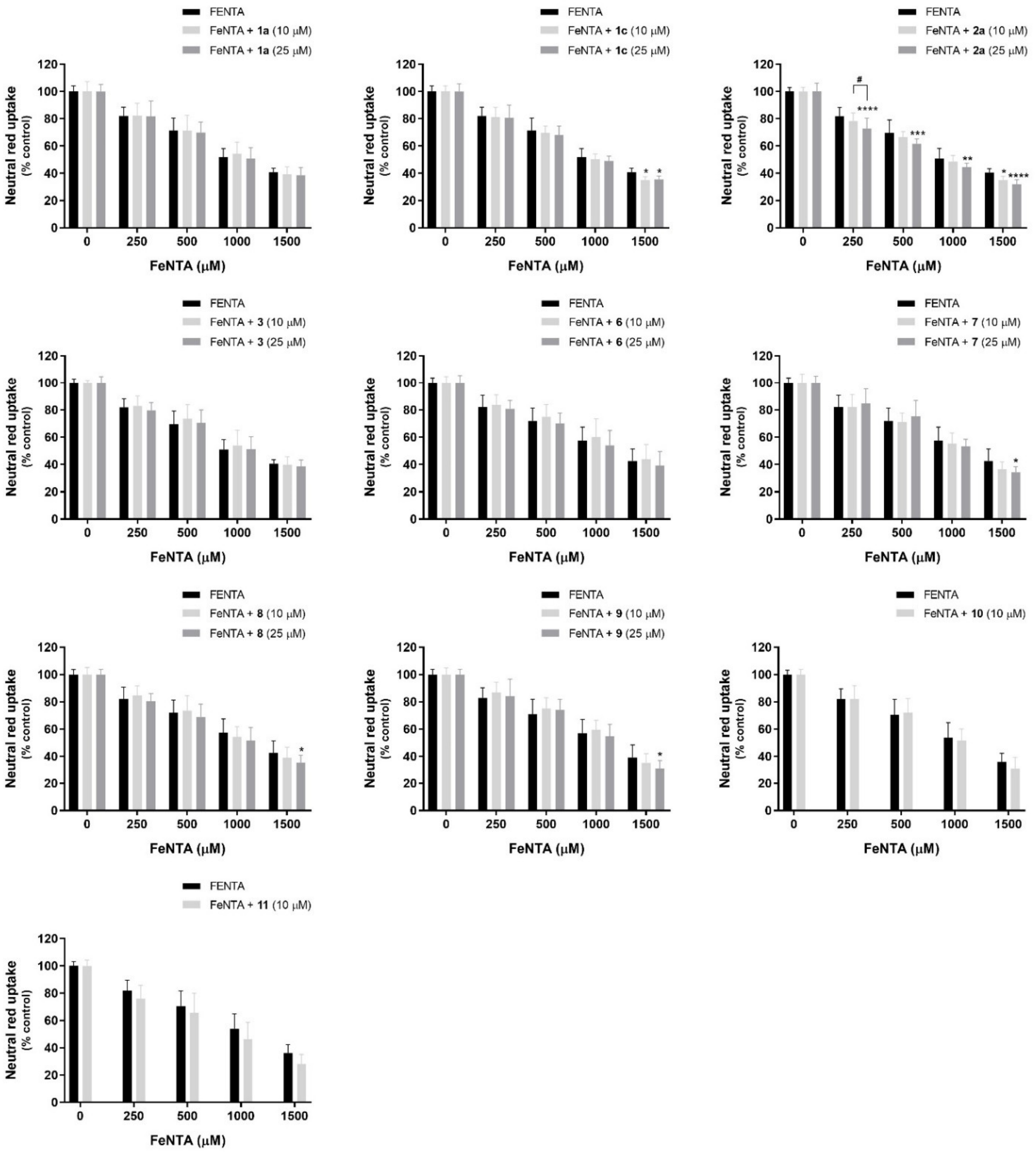

Figure S9. FeNTA (0 – 1500 μM) cytotoxicity evaluated in differentiated SH-SY5Y cells, in the presence or absence of fiscalins (10 and 25 μM), evaluated by the neutral red uptake assay, 48 hours after exposure. Results are presented as mean ± SD from 4 independent experiments, performed in triplicate. Statistical comparisons were made using two-way ANOVA, followed by Holm–Šídák's multiple comparisons test. In all cases, p values < 0.05 were considered significant [*$p < 0.05$; **$p < 0.001$; ***$p < 0.001$; ****$p < 0.0001$ *versus* FeNTA alone].

In the presence of fiscalin **1c**, at 10 and 25 μM, a small but significant decrease in the NR uptake was observed for 1500 μM of FeNTA (NR uptake significantly decreased to

35.0% and 35.5%, 48 hours after exposure to 1500 μM FeNTA in the presence of 10 and 25 μM of fiscalin **1c**, respectively, when compared to 40.7% for 1500 μM of FeNTA alone).

In the presence of fiscalin **2a** at 10 μM, a significant decrease in the NR uptake was also observed for 1500 μM of FeNTA (35.0%), when compared to FeNTA alone (40.5%). Additionally, a significant decrease in the NR uptake was also observed for all the tested concentrations of FeNTA in the presence of fiscalin **2a** at 25 μM, and when compared to FeNTA alone (NR uptake significantly decreased to 72.7%, 61.7%, 44.7% and 31.9%, 48 hours after exposure to 250, 500, 1000 and 1500 μM of FeNTA in the presence of 25 μM of fiscalin **2a**, respectively, and when compared to 81.9%, 69.5%, 51.0% and 40.5% of FeNTA alone).

Furthermore, after 48 hours of exposure of differentiated SH-SY5Y cells to FeNTA in the presence of compounds **7**, **8**, and **9** at 25 μM, a significant decrease in the NR uptake was observed for 1500 μM of FeNTA, and when compared to FeNTA alone (NR uptake significantly decreased to 34.3% and 35.5%, 48 hours after exposure to 1500 μM FeNTA in the presence of 25 μM of fiscalins **7** and **8**, respectively, when compared to 42.5% for 1500 μM of FeNTA alone; and NR uptake significantly decreased to 31.2%, 48 hours after exposure to 1500 μM FeNTA in the presence of 25 μM of fiscalin **9**, when compared to 39.3% for 1500 μM of FeNTA alone), further suggesting the potential of such compounds, at a noncytotoxic concentration, to aggravate the cytotoxicity induced by higher iron concentrations.
